# Identification of a novel family B DNA polymerase from *Enterococcus* phage IME199 and its overproduction in *Escherichia coli* BL21(DE3)

**DOI:** 10.1186/s12934-023-02228-6

**Published:** 2023-10-21

**Authors:** Pengjun Han, Huahao Fan, Yigang Tong

**Affiliations:** 1 College of Life Science and Technology, Beijing University of Chemical Technology, Beijing, 100029 China; 2 Beijing Advanced Innovation Center for Soft Matter Science and Engineering, Beijing University of Chemical Technology, Beijing, 100029 China

**Keywords:** IME199 DNA polymerase, *Escherichia coli* BL21 (DE3), Rolling circle amplification, Multiple displacement amplification

## Abstract

**Background:**

Identification and characterization of novel, faithful and processive DNA polymerases is a driving force in the development of DNA amplification methods. Purification of proteins from natural phages is often time-consuming, cumbersome and low yielding. *Escherichia coli* is a host bacterium widely used for the production of recombinant proteins, is the cell factory of choice for in vitro studies of phage protein function.

**Results:**

We expressed the gene encoding *Enterococcus faecium* phage IME199 DNA polymerase (IME199 DNAP) in *Escherichia coli* BL21(DE3), and characterized protein function. IME199 DNAP has 3′-5′ exonuclease activity, but does not have 5′-3′ exonuclease activity. In addition, IME199 DNAP has dNTP-dependent 5′-3′ polymerase activity and can amplify DNA at 15–35 °C and a pH range of 5.5–9.5. The amino acid residues Asp30, Glu32, Asp112 and Asp251 are the 3′-5′ exonuclease active sites of IME199 DNAP, while residues Asp596 and Tyr639 are essential for DNA synthesis by IME199 DNAP. More importantly, the IME199 DNAP has strand displacement and processive synthesis capabilities, and can perform rolling circle amplification and multiple displacement amplification with very low error rates (approximately 3.67 × 10^–6^).

**Conclusions:**

A novel family B DNA polymerase was successfully overproduced in *Escherichia coli* BL21(DE3). Based on the characterized properties, IME199 DNAP is expected to be developed as a high-fidelity polymerase for DNA amplification at room temperature.

**Supplementary Information:**

The online version contains supplementary material available at 10.1186/s12934-023-02228-6.

## Background

DNA polymerase is an important enzyme necessary for all living organisms and is responsible for DNA replication and repair [1]. Arthur Kornberg discovered the first DNA polymerase (DNA polymerase I) in *Escherichia coli* in 1955 [2], and subsequently a variety of DNA polymerases have been found in other prokaryotes and eukaryotes [3, 4]. The discovery of DNA polymerase largely contributed to our understanding of how DNA is replicated and repaired today, as well as to the creation and development of DNA amplification techniques.

Based on nucleotide sequence and structural similarity, DNA polymerases can be classified into seven families: A, B, C, D, X, Y and RT [5]. Family B DNA polymerases are relatively well-discovered, present in all domains of life, and have been reported in bacteria, archaea, eukaryotes, and viruses [6, 7]. Most family B DNA polymerases have at least two common domains: DNA polymerase domain and 3′-5′ exonuclease domain [8]. The family B DNA polymerases that have been reported can participate in genome replication in organisms using three different ways: replication using pre-existing nucleic acids (DNA or RNA) primers [9], protein- primed replication [10], and template-dependent de novo synthesis [11]. As the biochemical properties and functions of a large number of family B DNA polymerases have been investigated, their widespread application in molecular biology and biotechnology has been facilitated.

As the most abundant and widely distributed biological entities in the biosphere, bacteriophages (phages) have diverse genomes that also confer diversity in their DNA polymerase functions [12]. Phages that can encode their own DNA polymerase are constantly being discovered, and the DNA polymerases of phage phi29, T4, Bam35 and RB69 that have been characterized belong to the family B DNA polymerases [13–16]. Among all phage-encoded DNA polymerases, phi29 DNA polymerase (phi29 DNAP) is a model replicative DNA polymerase that has been extensively studied at the genetic, physiological and biochemical levels, and has been used in a variety of applications [17, 18]. The phi29 DNAP uses a protein as the primer to initiate genome replication, which is a paradigm for protein-primed replication. The high processivity and the capacity to couple polymerization to strand displacement endow phi29 DNA polymerase with powerful replication efficiency, which led to the development of its protocol for isothermal amplification in circular and linear genomic DNA. The development of rolling circle amplification (RCA) and multiple displacement amplification (MDA) methods has made phi29 DNA polymerase widely used in single-cell whole-genome amplification [19], genotyping of single nucleotide polymorphisms [20] and miRNA detection [21]. However, phi29 DNAP still has some limitations in its application, such as incomplete and uneven genome amplification and poor amplification of high G+C templates [22, 23]. The phi29 DNAP is only the tip of the iceberg of many phage DNA polymerases, and there are countless other promising DNA polymerases that need to be studied and developed, which will certainly provide new impetus to the field of molecular biology.

Phages rely on host bacteria for rapid reproduction, and it is very difficult to obtain phage-encoded proteins from the host after infection. Recombinant DNA expression technology in *Escherichia coli* (*E. coli*) provides assistance in studying functional proteins in phages. *E. coli* BL21 (DE3) has many advantages to support heterologous gene expression, such as increasing the production of soluble proteins, enhancing the formation of disulfide bonds, and facilitating the proper folding of target proteins [24], making it the first cell factory for heterologous production of phage proteins.

In our previous study, we isolated an *Enterococcus* phage IME199 with a total length of 18,838 bp [25]. Genome sequencing analysis revealed a 65 bp reverse repeat sequence at the end of the IME199 genome, which has the same properties as phi29-like phages [26]. In addition, the IME199 DNAP shares weak amino acid sequence similarity with phi29 DNAP, suggesting that they may be functionally similar, although IME199 and phi29 are very different taxonomically. In this study, we investigated the function of IME199 DNAP in terms of evolutionary analysis, protein production, and activity identification, and found that it has properties of high processivity, strand displacement, and high fidelity, which enable IME199 DNAP to perform RCA and MDA. This discovery enriches our understanding of phage DNA polymerase and provides a reference for the application of IME199 DNAP in the field of molecular biology.

## Results

### Phylogenetic analysis of IME199 DNAP

**Fig. 1:**
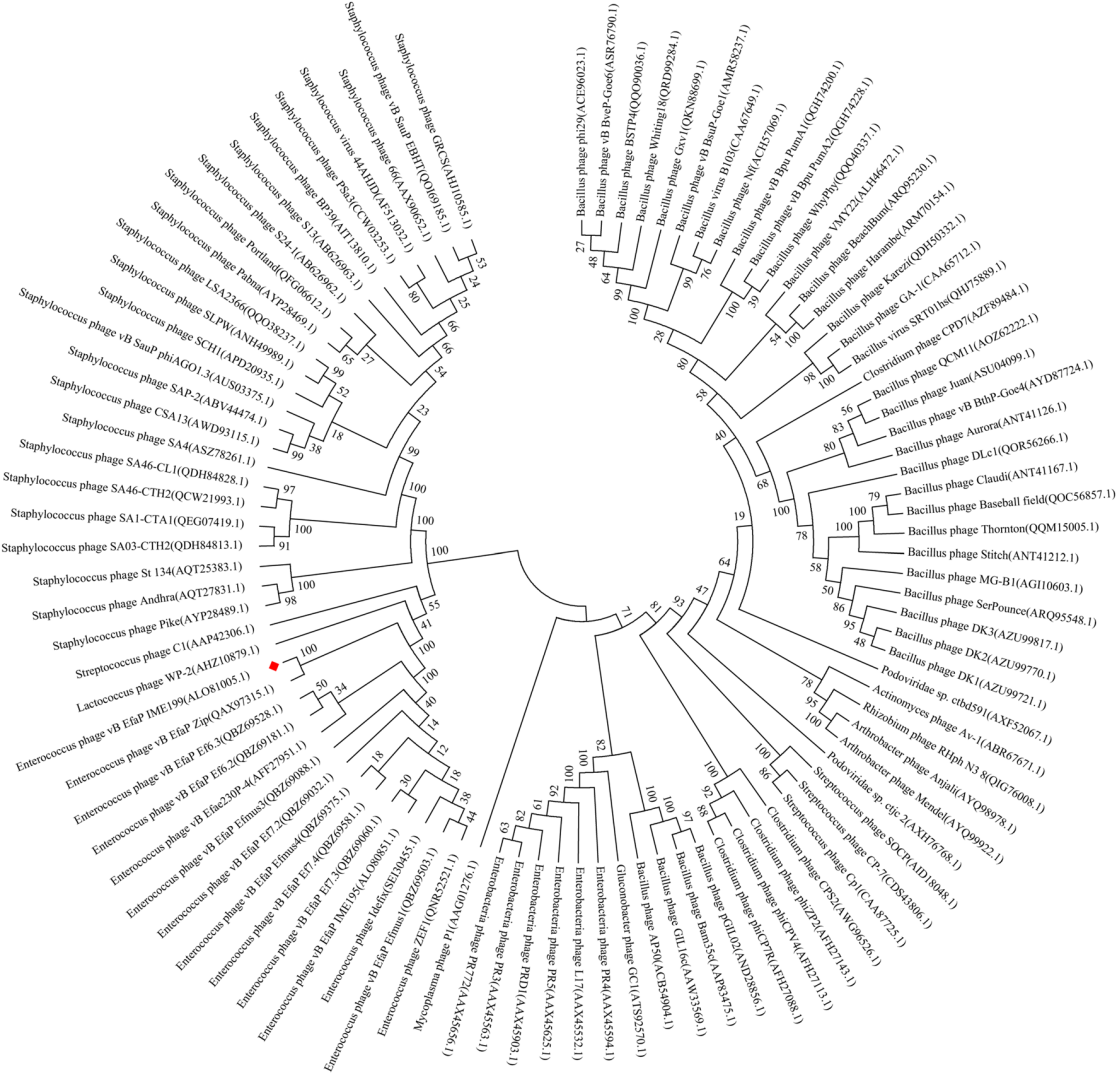
Phylogenetic analysis of IME199 DNAP. The phylogenetic tree was constructed based on the amino acid sequences of DNA polymerases using the Neighbor-joining method with 1000 bootstrap replicates in MEGA 7. All the amino acid sequences were downloaded from NCBI. IME199 DNAP is marked with a red square

Analysis of the amino acid sequence of IME199 DNAP (GenBank accession no. ALO81005.1) using InterPro [27] revealed that it has a DNA-directed DNA polymerase structural domain, which belongs to the family B DNA polymerases (Additional file 1: Fig. S1). To understand the evolutionary relationships of IME199 DNAP in homologous species, we constructed a phylogenetic tree based on amino acid sequence similarity. As shown in Fig. 1, we found that IME199 DNAP has some interrelationship with phage DNA polymerases of *Enterococcus*, *Streptococcus*, *Staphylococcus*, *Lactococcus*, *Clostridium and Bacillus*. Intriguingly, these different phage genomes have terminal inverted repeat sequences ranging from 6 to 378 bp (Additional file 1: Table S1). In the extensively studied phi29 like phages (during infection of *Bacillus*), terminal inverted repeat sequences are a prerequisite for protein-initiated DNA replication, allowing DNA polymerase to employ a sliding-back mechanism to recover information of the terminal nucleotides in genome replication [26, 28, 29]. Despite the lack of sequence similarity between the genomes of these phages which infect different genera of bacteria, their DNA polymerases may have important common functional and structural features. Amino acid sequence alignment of IME199 DNAP with other family B DNA polymerases showed that their 3′-5′ exonuclease active site is evolutionarily conserved which consists of three motifs (Exo I, Exo II, and Exo III), and that they also have conserved motifs associated with polymerase activity (Additional file 1: Fig. S2). The conserved motifs of family B DNA polymerases are essential for understanding the evolution of species [8], and are required for the biological functions of these enzymes. The evolutionary similarity of DNA polymerases and the features of inverted repeats at the end of the genome suggest that these phages may have the same replication mechanism and a common ancestor.

### IME199 DNAP possesses 3′-5′exonuclease activity

**Fig. 2:**
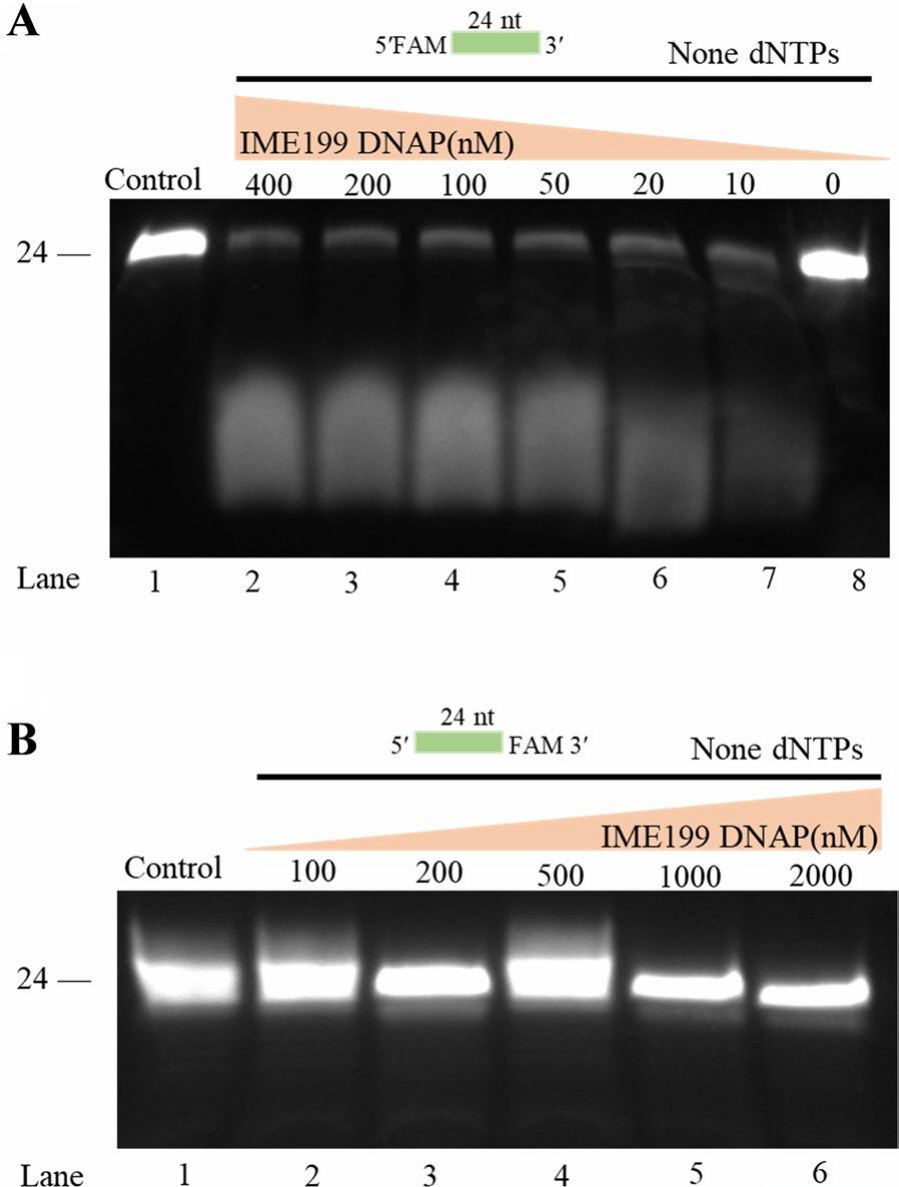
Exonuclease activity test of IME199 DNAP. Different concentrations of IME199 DNAP were incubated with 400 nM 5′-FAM-labeled 24 nt oligonucleotides (5′FAM-TCCTAACGAGATTAGTTTTGCTGT-3′) (**A**) or 3′-FAM-labeled 24 nt oligonucleotides (5′-TCCTAACGAGATTAGTTTTGCTGT -FAM3′) (**B**) at 30 °C for 20 min. The control is the FAM-labeled 24 nt oligonucleotide without IME199 DNAP. No deoxyribonucleoside triphosphates (dNTPs) were added in any of the reactions. All results are presented on 20% denaturing PAGE

To study the biochemical function of IME199 DNAP, its gene was cloned in pET-28a(+) and overexpressed in *E. coli* BL21(DE3), and the recombinant protein was purified after Ni-column affinity and gel filtration chromatography. Successfully produced and purified recombinant IME199 DNAP protein showed a band of approximately 95 kDa on SDS‒PAGE stained with Coomassie brilliant blue (Additional file 1: Fig. S3), which is consistent with the theoretical molecular mass of the amino acid sequence. We first examined the exonuclease activity of IME199 DNAP using modified ssDNA, and the results showed that IME199 DNAP has 3′-5′ exonuclease activity (Fig. 2A) but not 5′-3′exonuclease activity (Fig. 2B), which is typical of family B DNA polymerases.

DNA polymerase with 3′-5′ exonuclease activity plays an important role in promoting the accuracy of DNA replication and DNA proofreading and repair [30]. We found that IME199 DNAP with 3′-5′ exonuclease activity could remove the incorrect bases at the 3′ end of the primer that did not match the template, thus amplifying the correct product (Additional file 1: Fig. S4). This indicates that 3′-5′ exonuclease activity makes IME199 DNAP highly specific for inserting the correct base when performing replication and ensures high fidelity of DNA replication.

### DNA Polymerase activity of IME199 DNAP

**Fig. 3:**
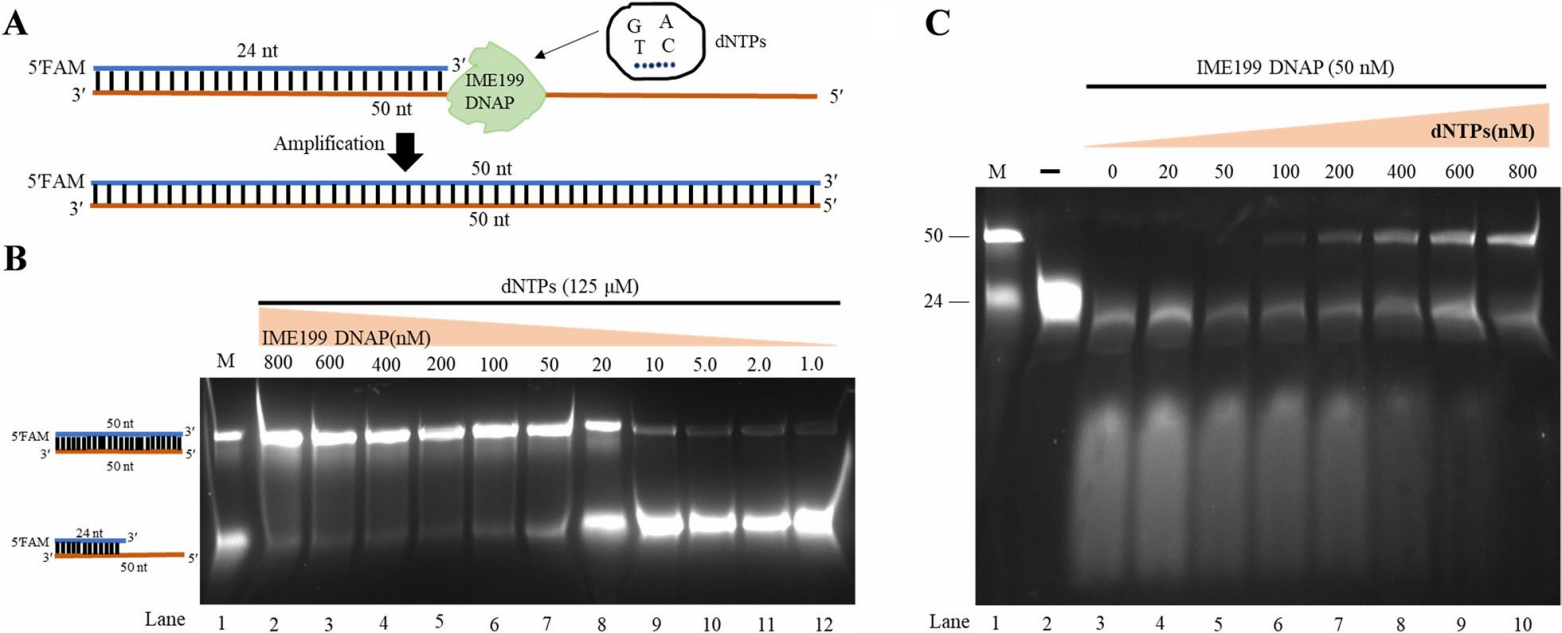
Polymerase activity test of IME199 DNAP. **A** Schematic representation of polymerase activity research. A primed-template (24/50 nt) substrate in which the primer was 5′-FAM–labeled was extended by IME199 DNAP into a complete double strand (50/50 nt) in the presence of dNTPs. **B** Extension of a primed-template (24/50 nt) substrate by IME199 DNAP. 400 nM substrate was incubated with different concentrations of IME199 DNAP and 125 μM dNTPs at 30 °C for 20 min, and then analyzed on a 20% denaturing PAGE gel. **C** Effect of dNTPs on IME199 DNAP polymerase activity. A primed-template (24/50 nt) substrate was incubated at 400 nM with 50 nM IME199 DNAP and different concentrations of dNTPs at 30 °C for 20 min, and then analyzed on a 20% denaturing PAGE gel. The primed-template (24/50 nt) substrate was made by hybridizing two oligonucleotide strands (24 nt oligonucleotide sequence: 5′FAM- TCCTAACGAGATTAGTTTTGCTGT -3′, 50 nt oligonucleotide sequence 5′-CCCATACAAATAAACCAAAAAACAATACAGCAAAACTAATCTCGTTAGGA-3′)

To evaluate the polymerase activity of IME199 DNAP, we analyzed the extension of labeled primer/template substrates (Fig. 3A). As shown in Fig. 3B, when dNTPs were sufficient, the yield of full-length products gradually increased with increasing IME199 DNAP concentration, and the products no longer increased after the IME199 DNAP concentration was greater than 50 nM. The 50 nM IME199 DNAP was incubated with the substrate in the presence of different concentrations of dNTPs, and the results showed that significant degradation products could be observed without total products in the absence or presence of low concentrations of dNTPs (< 100 nM) (Fig. 3C, lanes 3–5). In addition, the yield of full-length products gradually increased with increasing dNTPs concentration, and no degradation products were observed in the presence of 800 nM dNTPs (Fig. 3C, lanes 6–10).

### Biochemical characterization of IME199 DNAP

**Fig. 4:**
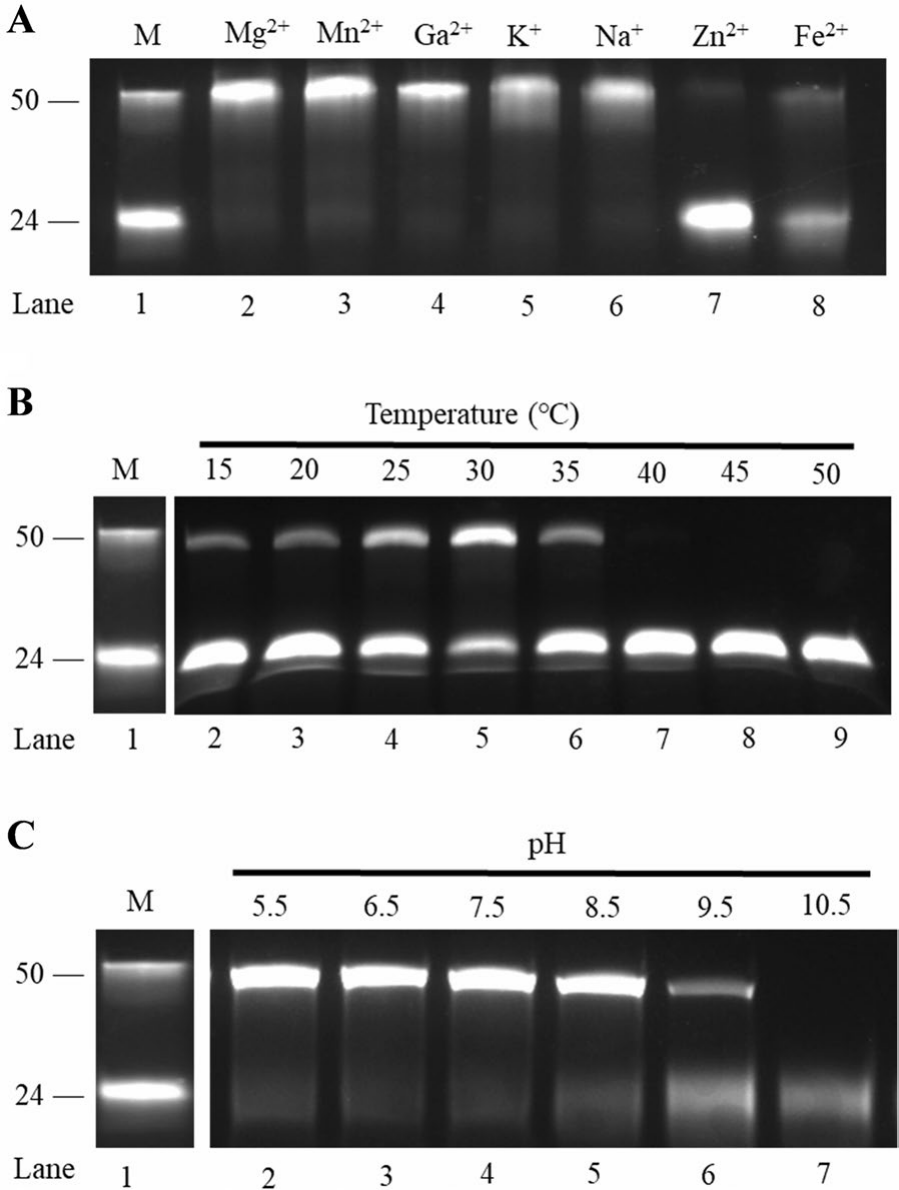
Biochemical characteristics of IME199 DNAP. **A** Effect of different metal ions on the polymerase activity of IME199 DNAP. 400 nM primed-template (24/50 nt) substrate was incubated with 50 nM IME199 DNAP and 125 μM dNTPs at 30 °C for 20 min in reaction buffers containing different metal ions (final concentration of 10 mM), and then analyzed on a 20% denaturing PAGE gel. **B** Effect of temperature on the polymerase activity of IME199 DNAP. 400 nM primed-template (24/50 nt) substrate was incubated with 50 nM IME199 DNAP and 125 μM dNTPs at different temperatures for 20 min in reaction buffers containing Mg^2+^, and then analyzed on a 20% denaturing PAGE gel. **C** Effect of different pH values on the polymerase activity of IME199 DNAP. 400 nM primed-template (24/50 nt) substrate was incubated with 50 nM IME199 DNAP and 125 μM dNTPs at 30 °C for 20 min in different pH reaction buffers, and then analyzed on a 20% denaturing PAGE gel. The primed-template (24/50 nt) substrate as same as Fig. 3

To determine the biochemical properties of DNA synthesized by IME199 DNAP, we examined the ability of IME199 DNAP to amplify labeled primer/template substrates in different metal cation buffers, different pH buffers and different temperatures. It is well known that replicative DNA polymerases require bivalent metal cations for polymerization. In this study, we found that IME199 DNAP could amplify full-length products in the presence of Mg^2+^, Mn^2+^, Ca^2+^, K^+^ or Na^+^ (Fig. 4A, lanes 2–6), whereas Zn^2+^ could not assist IME199 DNAP in the polymerization reaction. Studies on phage phi29 and Bam35 DNA polymerases have demonstrated that Mg^2+^ and Mn^2+^ can act as activators of B family DNA polymerases to promote the persistence of DNA replication [31, 32]. Unlike phi29 DNAP [33], Ca^2+^, K^+^ and Na^+^ can also act as metal activators of IME199 DNAP replication, but they are not as efficient as Mg^2+^ and Mn^2+^ in promoting polymerase activity.

Thermal stability tests showed that IME199 DNAP has polymerase activity in the range of 15–35 ℃, and 30 °C is the optimum reaction temperature (Fig. 4B, lanes 2–6). IME199 DNAP is sensitive to high temperatures and loses polymerase activity at 40 °C and above (Fig. 4B, lanes 7–9). The tolerance temperature range of IME199 DNAP is essentially close to room temperature, facilitating its development as a polymerase that does not require heating assistance for DNA amplification in the future.

Next, we investigated the effect of pH on the polymerase activity of IME199 DNAP. As shown in Fig. 4C, IME199 DNAP had good polymerase activity in the pH range of 5.5–8.5, and the polymerase activity decreased significantly when the pH was higher than 9.5.

### Mutational studies of IME199 DNAP

The tertiary structure of IME199 DNAP was predicted using SWISS-MODEL [34]. Although the similarity is less than 30%, the best reference model is the phi29 DNA polymerase orthorhombic crystal form (results not shown), indicating that IME199 DNAP and phi29 DNAP have structural similarities. Since the structure and active site of phi29 DNAP have been well studied, we compared the amino acid sequences of IME199 DNAP with those of phi29 DNAP and found that amino acid residues Asp30, Glu32, Asp112 and Asp251 of IME199 DNAP are similar to Asp12, Glu14, Asp66 and Asp169 of phi29 DNAP (Additional file 1: Fig. S5). These conserved amino acid residues are a part of the catalytic site of phi29 correspond to 3′-5′ exonuclease activity, and their mutation led to a 100-fold reduction in exonuclease activity [35, 36]. In addition, amino acid residues Tyr333, Gly335, Ser360, Thr575, Asp596 and Tyr639 of IME199 DNAP are also similar to Tyr226, Gly228, Ser252, Thr434, Asp458 and Tyr500 of phi29 DNAP (Additional file 1: Fig. S5), and these amino acid residues of phi29 DNAP have been shown to be essential for polymerase activity. Mutations of Tyr226 and Gly228 in phi29 DNAP can affect polymerase/exonuclease balance [37], and mutations of Ser252, Thr434, Asp458 and Tyr500 can affect in template-primer binding and cause loss of polymerase activity [38–42].

**Fig. 5:**
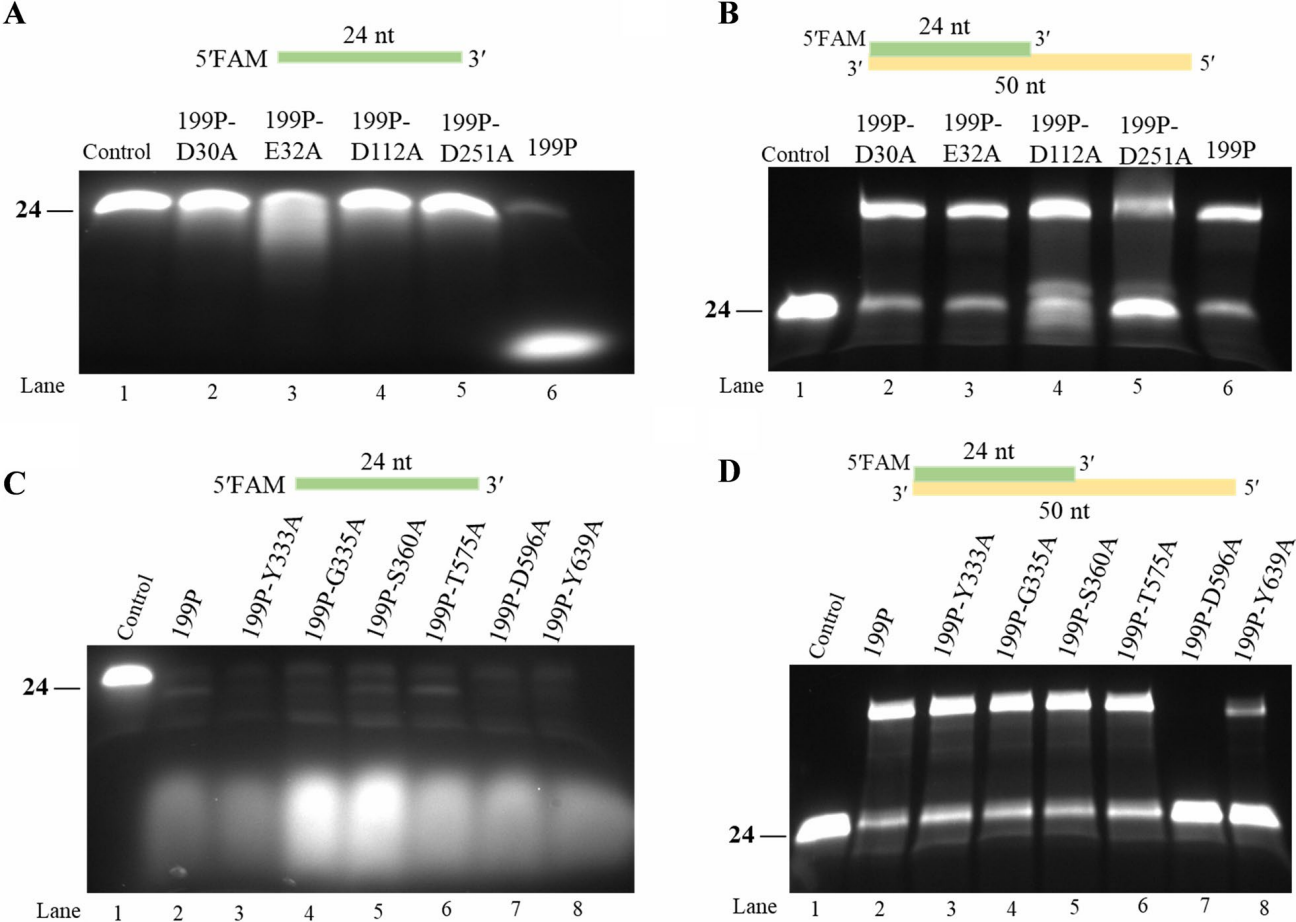
Study on the activity of IME199 DNAP mutant proteins. **A** Exonuclease activity test of IME199 DNAP and its mutant proteins (199P-D30A, 199P-E32A, 199P-D112A and 199P-D251A). The 5′-FAM-labeled 24 nt oligonucleotide (5′FAM- TCCTAACGAGATTAGTTTTGCTGT-3′) was incubated at 400 nM with 50 nM IME199 DNAP or its mutant proteins (199P-D30A, 199P-E32A, 199P-D112A and 199P-D251A) at 30 °C for 20 min and then analyzed on a 20% denaturing PAGE gel. **B** Polymerase activity test of IME199 DNAP and its mutant proteins (199P-D30A, 199P-E32A, 199P-D112A and 199P-D251A). The primed-template (24/50 nt) substrate was incubated at 400 nM with 50 nM IME199 DNAP or its mutant proteins (199P-D30A, 199P-E32A, 199P-D112A and 199P-D251A) and 125 μM dNTPs at 30 °C for 20 min, and then analyzed on a 20% denaturing PAGE gel. **C** Exonuclease activity test of IME199 DNAP and its mutant proteins (199P-Y333A, 199P-G335A, 199P-S360A, 199P-T575A, 199P-D596A and 199P-Y639A). The 5′-FAM-labeled 24 nt oligonucleotide (5′FAM- TCCTAACGAGATTAGTTTTGCTGT -3′) was incubated at 400 nM with 50 nM IME199 DNAP or its mutant proteins (199P-Y333A, 199P-G335A, 199P-S360A, 199P-T575A, 199P-D596A and 199P-Y639A) at 30 °C for 20 min and then analyzed on a 20% denaturing PAGE gel. (D) Polymerase activity test of IME199 DNAP and its mutant proteins (199P-Y333A, 199P-G335A, 199P-S360A, 199P-T575A, 199P-D596A and 199P-Y639A). The primed-template (24/50 nt) substrate was incubated at 400 nM with 50 nM IME199 DNAP or its mutant proteins (199P-Y333A, 199P-G335A, 199P-S360A, 199P-T575A, 199P-D596A and 199P-Y639A) and 125 μM dNTPs at 30 °C for 20 min, and then analyzed on a 20% denaturing PAGE gel. 199P represents IME199 DNAP in the figure

To confirm whether these similar amino acid residues are also essential for IME199 DNAP, we mutated 10 amino acids (Asp30, Glu32, Asp112, Asp251, Tyr333, Gly335, Ser360, Thr575, Asp596, Tyr639) of IME199 DNAP to alanine by targeted mutagenesis and tested the 3′-5′ exonuclease and polymerase activities of the mutant proteins. The results showed that the Asp30A, Glu32A, Asp112A and Asp251A mutant proteins lost 3′-5′ exonuclease activity (Fig. 5A, lanes 2–5) but still had polymerase activity (Fig. 5B, lanes 2–5), indicating that residues Asp30, Glu32, Asp112 and Asp251 are essential for IME199 DNAP 3′-5′ exonuclease activity. Meanwhile, the Tyr333A, Gly335A, Ser360A, Thr575A, Asp596A and Tyr639A mutant proteins still had 3′-5′ exonuclease activity (Fig. 5C, lanes 3–8), but only the Asp596A and Tyr639A mutant proteins almost lost polymerase activity (Fig. 5D, lanes 7 and 8), indicating that residues Asp596 and Tyr639 are essential for DNA synthesis by IME199 DNAP.

Amino acid residues mutational analysis of IME199 DNAP showed that the exonuclease structural domains of IME199 DNAP and phi29 DNAP are similar, but there are differences in their polymerase active structural domains, which also suggests that they are evolutionarily related. In addition, mutational analysis has also improved our understanding of the functional role of IME199 DNAP amino acid residues.

### Strand displacement and processive synthesis capability of IME199 DNAP for rolling circle amplification

**Fig. 6:**
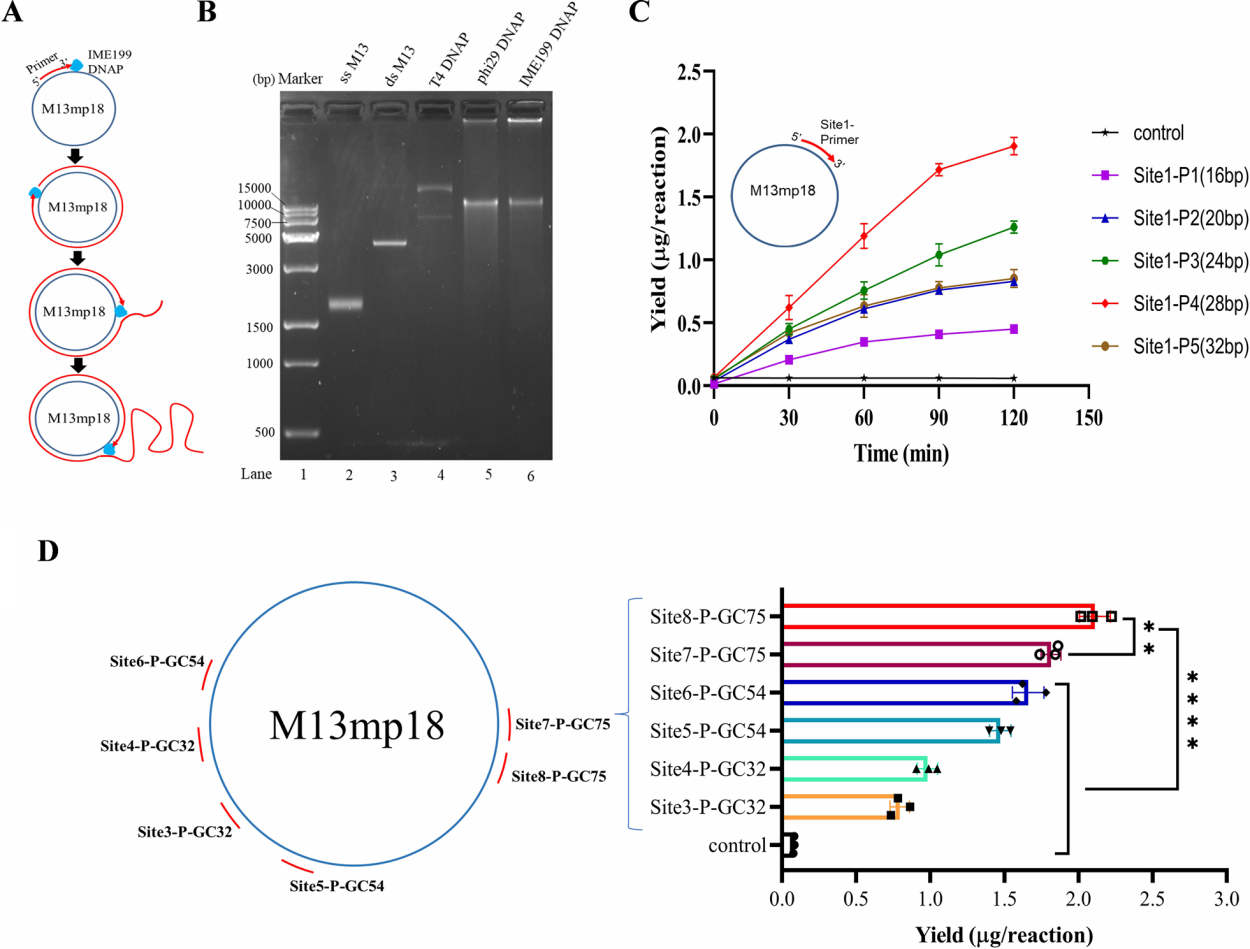
Rolling circle amplification of single-stranded circular M13mp18 by IME199 DNAP. **A** Schematic diagram of rolling circle amplification. DNA polymerase with strand displacement and processive synthesis capability amplifies primed M13mp18 DNA into a large single-stranded DNA product. **B** Agarose gel electrophoresis of IME199 DNAP, phi29 DNAP and T4 DNAP amplification primed M13mp18 products. The primed (primer sequence: 5′- TCGTAATCATGGTCATAGCTGTTTCCTG -3′) M13mp18 DNA was incubated at 25 ng with 10 U T4 DNAP, 10 U phi29 DNAP or 100 nM IME199 DNAP at 30 °C for 40 min, and then analyzed by non-denaturing 1% agarose electrophoresis. Lane 1, DNA marker; lane 2, M13mp18 single-stranded DNA; lane 3, M13mp18 double-stranded DNA; lane 4, T4 DNA polymerase; lane 5, phi29 DNA polymerase; lane 6, IME199 DNA polymerase. **C** Yield of M13mp18 amplified by IME199 DNAP using primers of different lengths. The assay was performed using 25 ng of single-stranded M13mp18 DNA and 100 nM IME199 DNAP. The primer sequences were listed in Additional file 1: Table S2. **D** Yield of M13mp18 amplified by IME199 DNAP using primers with different G+C content. 25 ng of single-stranded M13mp18 DNA was amplified by 100 nM IME199 DNAP at 30 °C for 1 h in the presence of primers with different G+C content. The primer sequences were listed in Additional file 1: Table S2. Data are shown as the mean ± SD. Statistical analysis was performed by one-way analysis of variance following a Dunnett’s multiple comparisons test. **P < 0.01, ****P < 0.0001

Strand displacement and processive synthesis are the outstanding characteristics of phi29 DNAP, making it widely used in the field of genetic engineering and biotechnology. Given the evolutionary and structural similarities between IME199 DNAP and phi29 DNAP, we hypothesized that IME199 DNAP also has strand displacement and processive synthesis capabilities. To verify this, we performed replication assay using the single-stranded circular DNA M13mp18 as a substrate (Fig. 6A). The results showed that IME199 DNAP can continuously amplify on the circular DNA template and produce a replication product larger than the full-length M13mp18 genome (7.25 kb), which is consistent with the product amplified by phi29DNAP (Fig. 6B, lanes 5–6). Some amplification products of IME199 DNAP and phi29 DNAP were larger compared to the amplification products of T4 DNA polymerase (T4 DNAP), which caused them to remain in the upper sample void. These results suggest that IME199 DNAP is similar to phi29 DNAP in its ability to couple strand displacement and processive polymerization during rolling circle DNA replication. Subsequently, we investigated the difference in different length primers for rolling loop amplification, and the results showed that IME199 DNAP amplified the most products using 28 bp primers (Fig. 6C), which was the same in different regions of the M13mp18 genome (Additional file 1: Fig. S6A). Under the same conditions, phi29 DNAP also had the highest yield using 28 bp primers (Additional file 1: Fig. S6B). However, we found that IME199 DNAP has a preference for high G+C primers when primer lengths are consistent. Significantly more products were amplified using high G+C (75%) primers than those using low G+C (≤ 54%) primers (Fig. 6D), while phi29DNAP did not have this preference under the same conditions (Additional file 1: Fig. S7).

### Fidelity of IME199 DNAP for multiple displacement amplification

**Fig. 7:**
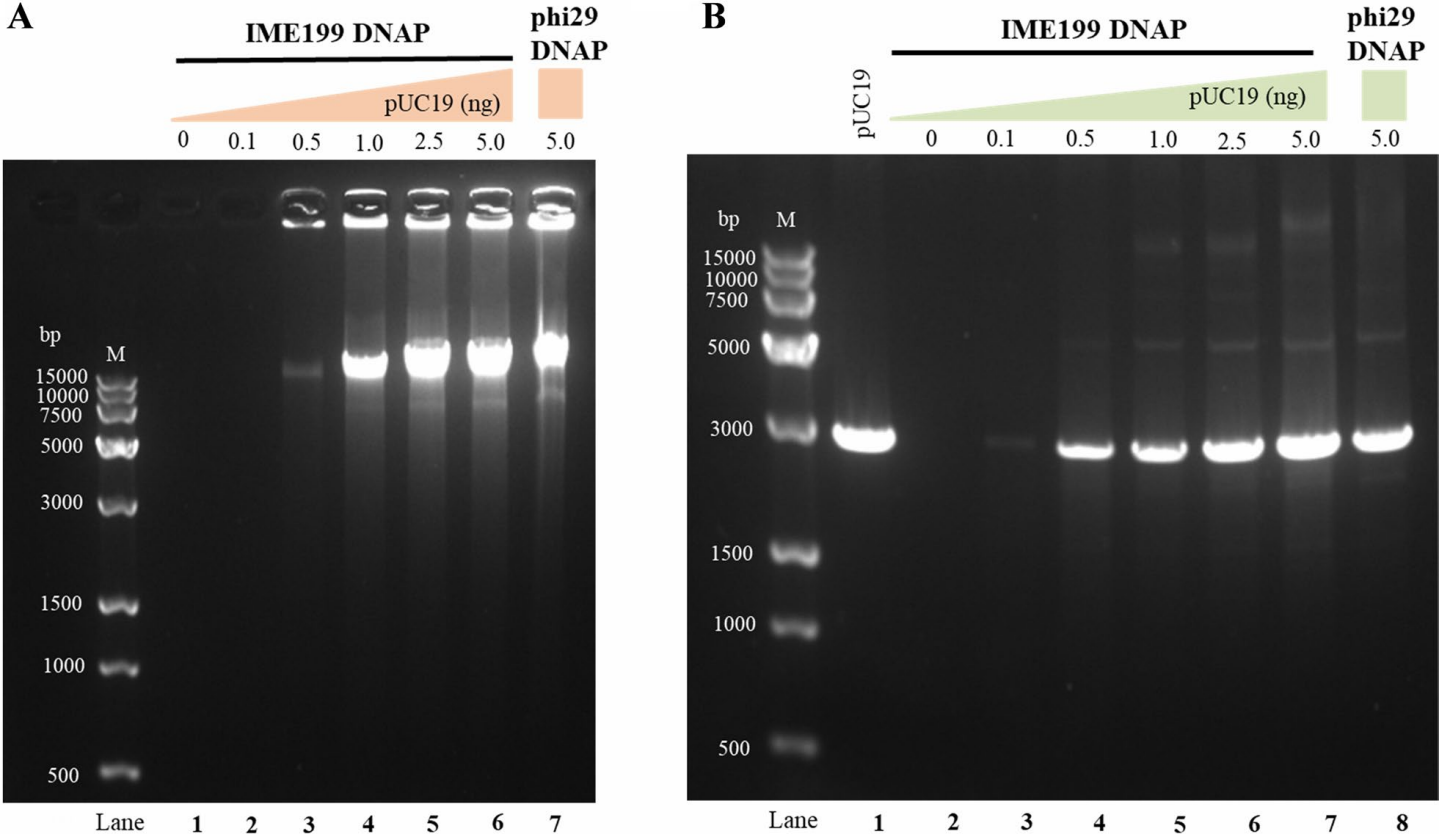
Multiple displacement amplification of pUC19 plasmid by IME199 DNAP. **A** The primed pUC19 DNA in which the heat-denatured pUC19 plasmid hybridized with random hexamer primers was incubated with 100 nM IME199 DNAP or 10 U phi29 DNAP (NEB, USA) at 30 °C for 2 h, and then analyzed by non-denaturing 1% agarose electrophoresis. **B** The pUC19 plasmid DNA and multiple displacement amplification of product by IME199 DNAP or phi29 DNAP was digested with SalI-HF for 2 h at 37 °C, and then analyzed by non-denaturing 1% agarose electrophoresis. Experimental details are described in Experimental Procedures

Phi29 DNAP can amplify DNA without sequence information using random hexamer primers, which is called multiple displacement amplification [43], and coupled with the high fidelity of amplification, phi29 DNAP has been widely used for whole genome amplification. We examined the amplification of the pUC19 plasmid by IME199 DNAP using random hexamer primers. The results showed that IME199 DNAP could amplify a product of the same size as phi29 DNAP using random hexamer primers (Fig. 7A, lanes 6 and 7). Simultaneous digestion of the amplified product and the pUC19 plasmid with endonuclease SalI followed by agarose gel electrophoresis analysis, the results showed that almost all of the amplified DNA was linearized after digestion, showing bands of the identical size as the digested pUC19 plasmid (approximately 2.7 kbp) (Fig. 7B, lanes 1–7), which was also the same as that of phi29 DNAP (Fig. 7B, lane 8). These results indicate that IME199 DNAP can be subjected to multiple displacement amplification and that the amplification products are tandem repeats of the template DNA.

Accurate DNA synthesis during replication is essential to maintain the stability of the genome over multiple generations [44]. The error rate of DNA replication is an important reflection of the fidelity of DNA polymerase, and a lower error rate represents higher fidelity. We determined the fidelity of IME199 DNAP for multiple displacement amplification using the blue‒white screening method [45] by exploiting the properties of the lacZα gene in the pUC19 plasmid. The average error rate of IME199 DNAP amplification was calculated to be 3.67 ± 0.55 × 10^−6^ by counting blue/white clones and detecting the yield of amplified products (Table 1), similar to that of phi29 DNAP and Bam35 DNAP, which was 5 × 10^−6^ [46] and 5.1 ± 1.7 × 10^–6^ [15], respectively. This result indicates that IME199 DNAP is also a high-fidelity DNA polymerase. Moreover, we sequenced the lacZα gene on the pUC19 plasmid in the mutant (white) clones and found that the mutation type of IME199 DNAP amplification was substitution and deletion, with the highest substitution rate and all C:G → T:A transitions (Additional file 1: Fig. S8).

**Table 1:** Determination of error rate values for IME199 DNAP during MDA

Experiment	mf	d	ER
1	6.9 × 10^−3^	6.78	3.1 × 10^−6^
2	9.3 × 10^−3^	6.81	4.2 × 10^−6^
3	8.2 × 10^−3^	6.86	3.7 × 10^−6^
Average ± SD	8.13 ± 1.20 × 10^−3^	6.82 ± 0.04	3.67 ± 0.55 × 10^−6^

The error rate was calculated using the equation ER = mf/(bp×d), where mf is the mutation frequency that was calculated using the equation mf = white colonies/total colonies, and bp is the number of detectable sites (324) in the lacZ gene of plasmid pUC19, and d is the number of template doublings that were calculated using the equation d = (ng product)/(ng input)

## Discussion

Over the past 60 years, the field of biotechnology has greatly benefited from the application of a large number of DNA polymerases with different properties. In order to fully develop and apply the functionality of novel DNA polymerases, systematic expression, purification and characterization processes are required. In this study, we constructed a heterologous expression system in *E. coli* for the production and purification of *Enterococcus* phage IME199 DNAP, and characterized the function of IME199 DNAP by biochemical methods. Evolutionary and structural domain analyses indicate that IME199 DNAP is a family B DNA polymerase, and as expected, IME199 DNAP possesses the basic activity characteristics of family B DNA polymerases: polymerase and 3′-5′ exonuclease activity. The 3′-5′ exonuclease activity of IME199DNAP allows it to remove mismatch bases from extended primers, thereby improving fidelity. The importance of proofreading activity for family B DNA polymerases has been demonstrated by the fact that their error rate increases more than tenfold when 3′-5′ exonuclease activity is inactivated [47, 48].

As a substrate for DNA polymerase, dNTPs are essential for DNA biosynthesis and repair, and they can regulate the polymerase activity and 3′-5′ exonuclease activity of DNA polymerase [49]. In this study, IME199 DNAP mainly exerted 3′-5′ exonuclease activity when the concentration of dNTPs was insufficient, while the 3′-5′ exonuclease activity of IME199 DNAP was replaced by polymerase activity when the concentration of dNTPs increased (Fig. 3C). IME199 DNAP was able to amplify substrates in the presence of low concentrations (100 nM) of dNTPs, which was generally consistent with the DNA polymerase of temperate phage Bam35 (phi29-like phage)[15]. However, different family B DNA polymerases exhibit different sensitivities to dNTPs. DNA polymerase from *Thermococcus gammatolerans* (Tga PolB) requires at least 2 μM dNTPs to amplify the substrate [50].

Metal ions are cofactors necessary for DNA polymerase to replicate DNA. Mg^2+^ and Mn^2+^ have been shown to be effective activators of family B DNA polymerase [50, 51]. Our results also indicate that Mg^2+^ and Mn^2+^ promote IME199DNAP replication capacity significantly better than other metal ions (Fig. 4A). DNA polymerase is sensitive to metal ion-induced changes in DNA conformation, which makes the ability of different metal ions to activate DNA polymerase to amplify DNA very different [52]. Although the amino acid sequences of IME199 DNAP and phi29 DNAP share many similar conserved motifs, their metal ion catalytic sites are different. Ca^2+^ activates DNA replication by IME199 DNAP, but is not an activator of phi29 DNAP. To date, only PabPolB (*Pyrococcus abyssi* GE5 DNA polymerase) in the family B DNA polymerases has been described to utilize Ca^2+^as a cofactor for DNA polymerization [53]. However, the rate of DNA synthesis using Ca^2+^ is low compared to Mg^2+^, so Mg^2+^ remains the preferred metal cofactor for DNA polymerase replication.

When DNA polymerase synthesizes deoxynucleoside triphosphate into nascent DNA, pyrophosphate and hydrogen ions are released, and the pH of the reaction system changes as the concentration of hydrogen ions increases. This change has made it possible to detect DNA amplification by using pH-sensitive indicator dyes, which has been used in loop mediated isothermal amplification (LAMP) and rolling circle amplification (RCA) [54, 55]. In this study, IME199 DNAP has been shown to be effective for rolling circle amplification while exhibiting stable polymerase activity at pH 5.5–9.5. Based on this property, the amplification reaction of IME199 DNAP can be visually detected using weakly alkaline pH indicators (results not shown), which provides a possibility for the application of IME199 DNAP in isothermal amplification detection in the future.

DNA polymerases with strand displacement activity, such as phi29 DNAP, Bst DNAP and Bsu DNAP, are core to isothermal amplification technology, and these enzymes are able to displace downstream DNA during replication, thus eliminating the need for thermal denaturation [56–58]. Our study confirmed that IME199 DNAP has similar strand replacement and processive synthesis capabilities as phi29 DNAP. Unlike phi29 DNAP, IME199 DNAP has better amplification effect using high G+C primers, and the yield of amplifying high G+C sequences is also higher than that of amplifying low G + C sequences under the same conditions (Additional file 1: Fig. S9). This property could enable IME199 DNAP to be used to amplify high G+C content DNA templates, such as soil microorganisms with high G+C genomes [59]. In addition, based on the characteristics of RCA, IME199 DNAP could be used for point-of-care testing of circular DNA viruses (such as Transfusion Transmitted viruses and Norovirus).

## Conclusion

In summary, we overproduced a DNA polymerase from *Enterococcus faecium* phage IME199 in *E. coli* BL21 (DE3), and described its biological properties. Evolutionary analysis and mutation studies demonstrated that IME199 DNAP has great similarity to the extensively studied phi29 DNAP. IME199 DNAP has both 3′-5′ exonuclease activity and polymerase activity, which are essential properties of family B DNA polymerases. IME199 DNAP has the ability for strand displacement and processive synthesis, and can perform rolling circle amplification and multiple displacement amplification at 30 °C similar to phi29 DNAP. However, unlike phi29 DNAP, Ca^2+^ ions can act as metal activators to assist IME199 DNAP in DNA replication. Meanwhile, IME199 DNAP can amplify more DNA products using high G+C primers or on high G+C templates. Our study provides an important reference for the further development of IME199 DNAP and its application in isothermal amplification.

## Experimental procedures

### Phylogenetic analyses of IME199 DNAP

The amino acid sequences of IME199 DNAP and other phage polymerases were obtained from the National Center for Biotechnology Information (NCBI), and the phylogenetic tree was constructed with MEGA7 using the Neighbour-Joining algorithm [60].

### Protein production and purification

The sequences of IME199 DNAP were amplified by PCR from bacteriophage IME199 genomic DNA. The target sequence was constructed into the pET-28a ( +) vector using BamHI/XhoI enzyme cutting sites, and the protein was expressed in *E. coli* BL21 (DE3) as described elsewhere [61] with some modifications. In brief, *E. coli* BL21(DE3) cells harboring the recombinant plasmids were cultured in LB medium containing 50 μg/mL kanamycin at 37 °C until they reached an OD600 of 0.6. Then, target proteins were induced by adding 0.1 mM isopropyl β-D-thiogalactoside (IPTG), and the cells continued to be cultured for 20 h at 16 °C. Subsequently, cells were collected by centrifuging at 10,000 × g for 10 min, and resuspended in buffer A (20 mM Tris–HCl, 500 mM NaCl, 10% glycerol, pH 7.4). Further protein purification steps were carried out using a His-tag Protein Purification Kit (Beyotime, China) according to the manufacturer’s instructions. The target proteins were further purified by size exclusion chromatography via a Superdex 200 gel filtration column (GE Healthcare Life Sciences China) with buffer B (10 mM HEPES, 150 mM NaCl, 5% glycerol, pH 7.4). The effluent containing target proteins was pooled and concentrated using an Amicon Ultra-50 centrifugal filter unit. After examining its purity by 10% (w/v) sodium dodecyl sulfate‒polyacrylamide gel electrophoresis (SDS‒PAGE), the IME199 DNAP was maintained in the presence of 50% glycerol at − 20 °C for storage.

In addition, protein variants of IME199 DNAP (D30A, E32A, D112A, D251A, Y333A, G335A, S360A, T575A, D596A, Y639A) were constructed using the Fast Mutagenesis System (Cat# FM111-01, TransGen Biotech, Beijing, China) according to the manufacturer’s instructions, and these proteins were expressed and purified using the same method as described above. All primers used in this study are listed in Additional file 1: Table S2.

### Exonuclease and polymerase activity assay

The exonuclease and polymerase activity assay were performed by using oligonucleotides as previously described [62] with minor modifications. The 5′-FAM-labeled 24 nt oligonucleotides ENC-1 (5′- TCCTAACGAGATTAGTTTTGCTGT -3′) and the 3′-FAM-labeled 24 nt oligonucleotides ENC-2 (5′- TCCTAACGAGATTAGTTTTGCTGT -3′) were used as substrates for the exonuclease activity test. Different concentrations of IME199 DNAP or mutant proteins were incubated with 400 nM oligonucleotides ENC-1 or ENC-2, 50 mM Tris–HCl, 10 mM (NH_4_)_2_SO_4_, 4 mM DTT and 10 mM MgCl_2_ in a final volume of 20 μL (pH 7.5) at 30 °C for 20 min.

Oligonucleotides ENC-1 were hybridized to PLM-R (5′- CCCATACAAATAAACCAAAAAACAATACAGCAAAACTAATCTCGTTAGGA -3′) forming a primer/template structure for the polymerase activity test. Different concentrations of IME199 DNAP or mutant proteins were incubated with 400 nM primer/template, 50 mM Tris–HCl, 10 mM (NH_4_)_2_SO_4_, 10 mM MgCl_2_, 4 mM DTT and different concentrations of dNTPs in a final volume of 20 μL (pH 7.5) at 30 °C for 20 min.

Finally, a 20% denaturing PAGE gel was prepared using 20% Urea-PAGE electrophoresis kit (Coolaber, Beijing, China) and used to separate the reaction products under electrophoretic conditions at 30 mA for 50 min. Then, the gel was visualized using fluorescence imaging with the Tanon Imaging System (Tanon 5200 Multi, Biotanon, China). All oligonucleotides were synthesized from Beijing Rui Biotech Co., Ltd and listed in Additional file 1: Table S2.

### Optimization of the polymerase activity of IME199 DNAP

To investigate the effects of different metal ions on IME199 DNAP polymerase activity, 10 mM MgCl_2_, MnCl_2_, CaCl_2_, KCl, NaCl, ZnCl_2_ or FeSO_4_ was added to 20 μL of reaction mixture (50 nM IME199 DNAP, 400 nM primer/template, 50 mM Tris–HCl, 10 mM (NH_4_)_2_SO_4_, 125 μM dNTPs, 4 mM DTT, pH 7.5) individually and incubated at 30 °C for 20 min. The results were visualized as described above.

For the optimal temperature assay, the reaction mixture (20 μL, pH 7.5) contained 50 nM IME199 DNAP, 400 nM primer/template, 50 mM Tris–HCl, 10 mM (NH_4_)_2_SO_4_, 10 mM MgCl_2_, 125 μM dNTPs, 4 mM DTT were incubated at 15 °C, 20 °C, 25 °C, 30 °C, 35 °C, 40 °C and 45 °C for 20 min, respectively. The results were visualized as described above.

To determine the optimal pH, 50 nM IME199 DNAP, 400 nM primer/template, 10 mM (NH_4_)_2_SO_4_, 10 mM MgCl_2_, 125 μM dNTPs and 4 mM DTT were added into the Tris buffers with different pH values ranging from 5.5 to 10.5, and the reaction mixture (20 μL) were incubated at 30 °C for 20 min. The results were visualized as described above.

### Strand displacement and rolling circle amplification analysis

Strand displacement assay using single-stranded circular M13mp18 (NEB, USA) as the template. The assay was carried out in the presence of 100 nM IME199 DNAP or 10 U phi29 DNAP (NEB, USA) or 10U T4 DNAP (NEB, USA), 10 mM MgCl_2_, 50 mM Tris–HCl, 10 mM (NH_4_)_2_SO_4_, 250 μM dNTPs, 4 mM DTT, 25 ng M13mp18 (NEB, USA) and 1 μM primer Site1-P4 (5′-TCGTAATCATGGTCATAGCTGTTTCCTG -3′) in a 25 μL reaction. After incubation for the indicated times at 30 °C, the reaction was stopped by adding 25 mM EDTA and 0.5% SDS. DNA replication products were analyzed by electrophoresis in 1.0% agarose contained Genecolour II nucleic acid dyes under electrophoretic conditions at 120 V for 50 min, and the gel was analyzed using fluorescence imaging with the Tanon Imaging System (Tanon 5200 Multi, Biotanon, China).

In addition, the characteristics of IME 199DNAP were investigated in RCA using different primers with M13mp18 (NEB, USA) as the substrate according to the above method. The concentration of DNA was measured using the DNA HS Assay Kit (Cat# FS-T1002, Beijing Foreverstar Biotech Co., Ltd, China) according to the manufacturer’s instructions. The yield of DNA was calculated using the equation Yield = v × c, where v is the reaction volume and c is the concentration of detected DNA.

### Multiple displacement amplification and fidelity analysis

Multiple displacement amplification assay was performed using the plasmid pUC19 (NEB, USA) as substrate. In brief, the pUC19 plasmid (NEB, USA) was mixed with random hexamer primers (NEB, USA) and incubated at 95 °C for 3 min and cooled to room temperature. The formed primed pUC19 DNA was added to the reaction mixture (25 μL, pH 7.5) contained 100 nM IME199 DNAP or 10 U phi29 DNAP (NEB, USA), 50 mM Tris -HCl, 10 mM (NH_4_)_2_SO_4_, 10 mM MgCl_2_, 500 μM dNTPs, 4 mM DTT, and incubated at 30 °C for 2 h. Electrophoresis and analysis were performed as described above.

The fidelity of IME199 DNAP was measured as previously described [15] with some modifications. In brief, IME199 DNAP or phi29 DNAP was incubated with 1 ng pUC19 (NEB, USA) at 30 °C for 8 h according to the above system. Amplification products were digested with restriction endonuclease SalI-HF (NEB, USA), heat inactivated, ligated, transformed into XL-10 competent cells (Vazyme, China), and plated onto Luria–Bertani solid plates containing ampicillin (50 μg/mL), X-Gal (0.8 mg) and IPTG (0.8 mg). Plates were incubated at 37 °C for 16 h and then the blue and white colonies were counted. In addition, the plasmids extracted from the white clones were sent to Ruibiotech (Beijing, China) for sequencing to identify the mutation site.

### Supplementary Information

**Additional file 1: Figure S1**. Structural domain prediction using InterPro for IME199 DNAP. IME199 DNAP has a DNA-dir_DNA_pol_B_mt/vir (IPR004868) structural domain, which belongs to the typical DNA polymerase B family. **Figure S2**. Alignment of the IME199 DNAP amino acid sequence with other family B DNA polymerases. The alignment was generated by MAFFT [1] and ESPript [2]. IME199 DNAP: *Enterococcus* phage IME199 DNA polymerase (GenBank accession no. ALO80851.1); phi29 DNAP: *Bacillus* phage phi29 DNA polymerase (GenBank accession no. ACE96023.1); 44AHDJ DNAP: *Staphylococcus* phage phi44AHJD DNA polymerase (GenBank accession no. AF513032.1); C1 DNAP: *Streptococcus* phage C1 DNA polymerase (GenBank accession no. AAP42306.1); WP-2 DNAP: *Lactococcus* phage WP-2 DNA polymerase (GenBank accession no. AHZ10879.1); CPS2 DNAP: *Clostridium* phage CPS2 DNA polymerase (GenBank accession no. AWG96526.1). Identical amino acid residues on a red background. The conserved motif sequences are underlined in black. **Figure S3**. Coomassie brilliant blue stained SDS-PAGE (10%) showing purified wild-type IME199 DNAP and its mutants (~95 kDa). The same protein size marker (Blue Plus^®^ V, TransGen, China) was used in the experiment. All purified recombinant proteins have a 6-His label on the N-terminal. 199P represents IME199 DNAP in the figure. **Figure S4**. Study on the correction function of IME199 DNAP. IME199 DNAP was incubated with primed-templates mismatched 3′-terminus for 20 min at 30°C and then analyzed on a 20% denaturing PAGE gel. “−” means no IME199 DNAP, and “+” means with IME199 DNAP. The primed-template (25/50 nt) substrate was made by hybridizing two oligonucleotide strands (25 nt oligonucleotide sequence: 5′FAM- TCCTAACGAGATTAGTTTTGCTGTT -3′, 50 nt oligonucleotide sequence 5′- CCCATACAAATAAACCAAAAAACAATACAGCAAAACTAATCTCGTTAGGA -3′). The primed-template (27/50 nt) substrate was made by hybridizing two oligonucleotide strands (27 nt oligonucleotide sequence: 5′FAM- TCCTAACGAGATTAGTTTTGCTGTTAA -3′, 50 nt oligonucleotide sequence 5′- CCCATACAAATAAACCAAAAAACAATACAGCAAAACTAATCTCGTTAGGA -3′). The primed-template (29/50 nt) substrate was made by hybridizing two oligonucleotide strands (29 nt oligonucleotide sequence: 5′FAM- TCCTAACGAGATTAGTTTTGCTGTTAACA -3′, 50 nt oligonucleotide sequence 5′- CCCATACAAATAAACCAAAAAACAATACAGCAAAACTAATCTCGTTAGGA -3′). **Figure S5**. Amino acid sequence analysis between IME199 DNAP and phi29 DNAP. Alignment between IME199 DNAP and phi29 DNAP was made using CLC Sequence Viewer 6, and the regions containing homologous sequences are shown. Numbers indicate amino acid sites. Green color indicates amino acid residues of IME199 DNAP that are similar to those of phi29 DNAP. The conserved motif sequences are underlined in black. Red stars indicate amino acid residues associated with 3′-5′ exonuclease activity in phi29 DNAP. Pink diamond indicates amino acid residues associated with polymerase activity in phi29 DNAP. **Figure S6**. The yield of rolling circle amplification. 25 ng of single-stranded M13mp18 DNA was amplified by 100 nM IME199 DNAP (**A**) or 10 U phi29 DNAP (**B**) at 30 °C in the presence of primers of different lengths. The yield of DNA was calculated using the equation Yield = v × c, where v is the reaction volume and c is the concentration of detected DNA. Primer sequences are shown in Table S2. **Figure S7**. Comparison of phi29 DNAP using 28bp primers with different G+C content for rolling circle amplification. 25 ng of single-stranded M13mp18 DNA was amplified by 10 U phi29 DNAP at 30 °C in the presence of primers with different G+C content. The yield of DNA was calculated using the equation Yield = v × c, where v is the reaction volume and c is the concentration of detected DNA. Primer sequences are shown in Table S2. **Figure S8**. Sequencing results of the lacZα gene on the pUC19 plasmid in the white clones. On the left is the lacZ gene sequence, and the numbers represent the base sites. On the right is a summary of the sequencing results of the pUC19 plasmid in 26 white clones. **Figure S9**. Comparison of IME199 DNAP amplified different G+C sequences. Amplification was performed at 30 ℃ using random hexamer primers. Gene1 and gene2 are linear double-stranded DNA with a length of about 1600bp. **Table S1.** Analysis of phage genomes with terminal inverted repeat sequences. **Table S2: **Sequences of the oligonucleotides that have been used in this study.

## Data Availability

All data for this study are included in this article and its supplementary material file.
