# Supplementary material for: Identification of a novel family B DNA polymerase from Enterococcus phage IME199 and its overproduction in Escherichia coli BL21(DE3)
Source: Microb Cell Fact. 2023 Oct 21;22:217. doi: 10.1186/s12934-023-02228-6 (PMC10590003; doi:10.1186/s12934-023-02228-6)
Supplement: Supplementary file 1 — Additional file 1: Figure S1. Structural domain prediction using InterPro for IME199 DNAP. IME199 DNAP has a DNA-dir_DNA_pol_B_mt/vir (IPR004868) structural domain, which belongs to the typical DNA polymerase B family. Figure S2. Alignment of the IME199 DNAP amino acid sequence with other family B DNA polymerases. The alignment was generated by MAFFT [1] and ESPript [2]. IME199 DNAP: Enterococcus phage IME199 DNA polymerase (GenBank accession no. ALO80851.1); phi29 DNAP: Bacillus phage phi29 DNA polymerase (GenBank accession no. ACE96023.1); 44AHDJ DNAP: Staphylococcus phage phi44AHJD DNA polymerase (GenBank accession no. AF513032.1); C1 DNAP: Streptococcus phage C1 DNA polymerase (GenBank accession no. AAP42306.1); WP-2 DNAP: Lactococcus phage WP-2 DNA polymerase (GenBank accession no. AHZ10879.1); CPS2 DNAP: Clostridium phage CPS2 DNA polymerase (GenBank accession no. AWG96526.1). Identical amino acid residues on a red background. The conserved motif sequences are underlined in black. Figure S3. Coomassie brilliant blue stained SDS-PAGE (10%) showing purified wild-type IME199 DNAP and its mutants (~95 kDa). The same protein size marker (Blue Plus® V, TransGen, China) was used in the experiment. All purified recombinant proteins have a 6-His label on the N-terminal. 199P represents IME199 DNAP in the figure. Figure S4. Study on the correction function of IME199 DNAP. IME199 DNAP was incubated with primed-templates mismatched 3′-terminus for 20 min at 30°C and then analyzed on a 20% denaturing PAGE gel. “−” means no IME199 DNAP, and “+” means with IME199 DNAP. The primed-template (25/50 nt) substrate was made by hybridizing two oligonucleotide strands (25 nt oligonucleotide sequence: 5′FAM- TCCTAACGAGATTAGTTTTGCTGTT -3′, 50 nt oligonucleotide sequence 5′- CCCATACAAATAAACCAAAAAACAATACAGCAAAACTAATCTCGTTAGGA -3′). The primed-template (27/50 nt) substrate was made by hybridizing two oligonucleotide strands (27 nt oligonucleotide sequence: 5′FAM- TCCTAACGAGATTAGTTTTGCTGT [file 12934_2023_2228_MOESM1_ESM.docx]

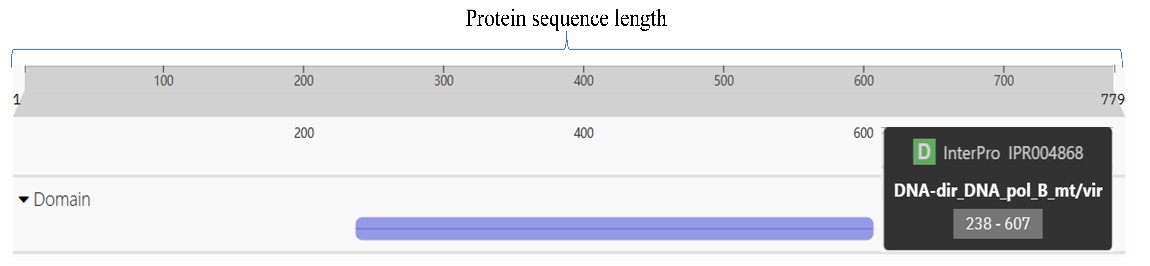


**Additional file 1: Figure S1**. Structural domain prediction using InterPro for IME199 DNAP. IME199 DNAP has a DNA-dir_DNA_pol_B_mt/vir (IPR004868) structural domain, which belongs to the typical DNA polymerase B family


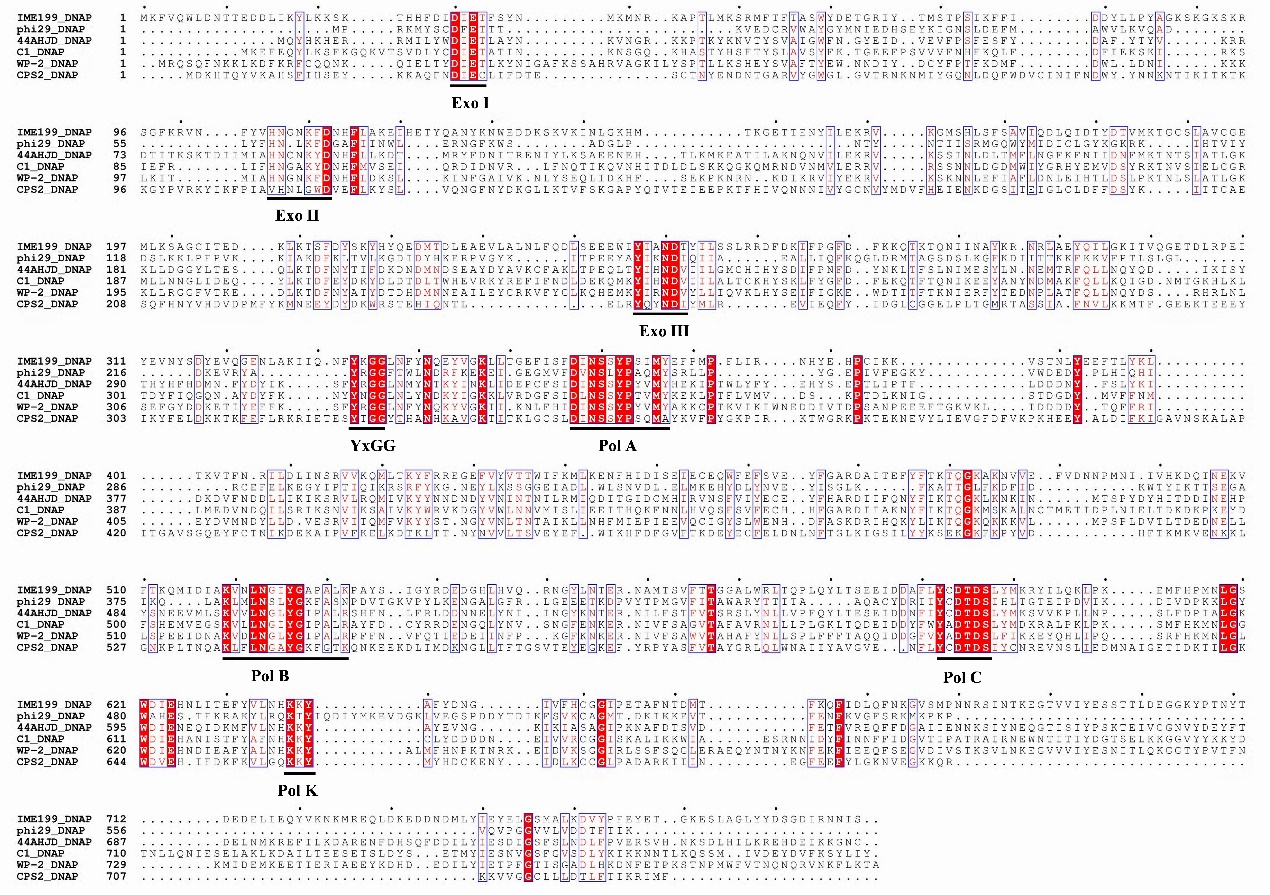


**Additional file 1: Figure S2**. Alignment of the IME199 DNAP amino acid sequence with other family B DNA polymerases. The alignment was generated by MAFFT^[1]^ and ESPript^[2]^. IME199 DNAP: *Enterococcus* phage IME199 DNA polymerase (GenBank accession no. ALO80851.1); phi29 DNAP: *Bacillus* phage phi29 DNA polymerase (GenBank accession no. ACE96023.1); 44AHDJ DNAP: *Staphylococcus* phage phi44AHJD DNA polymerase (GenBank accession no. AF513032.1); C1 DNAP: *Streptococcus* phage C1 DNA polymerase (GenBank accession no. AAP42306.1); WP-2 DNAP: *Lactococcus* phage WP-2 DNA polymerase (GenBank accession no. AHZ10879.1); CPS2 DNAP: *Clostridium* phage CPS2 DNA polymerase (GenBank accession no. AWG96526.1). Identical amino acid residues on a red background. The conserved motif sequences are underlined in black.


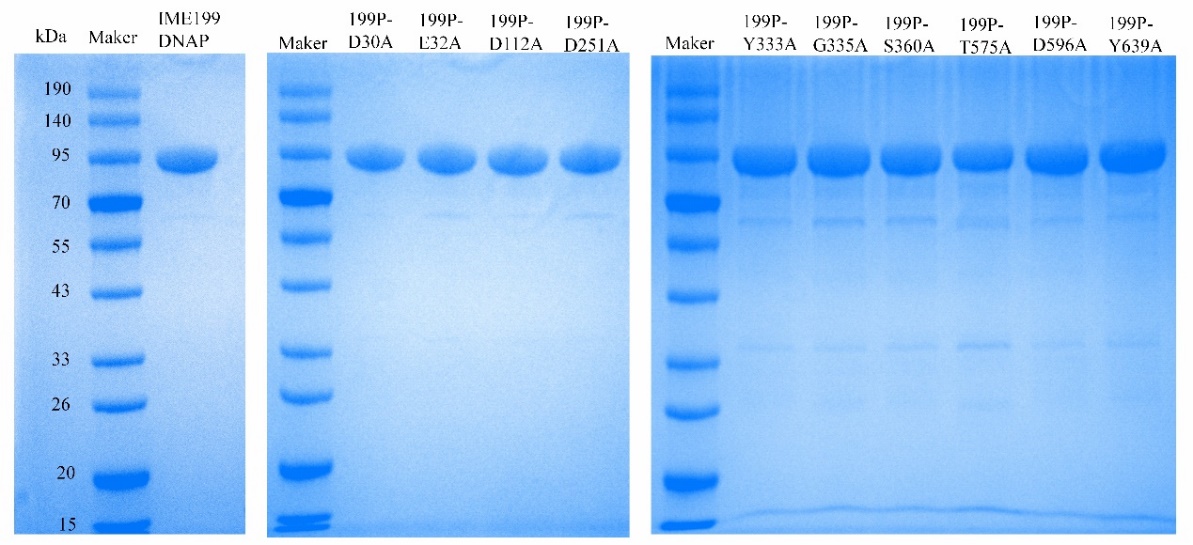


**Additional file 1: Figure S3**. Coomassie brilliant blue stained SDS-PAGE (10%) showing purified wild-type IME199 DNAP and its mutants (~95 kDa). The same protein size marker (Blue Plus® V, TransGen, China) was used in the experiment. All purified recombinant proteins have a 6-His label on the N-terminal. 199P represents IME199 DNAP in the figure


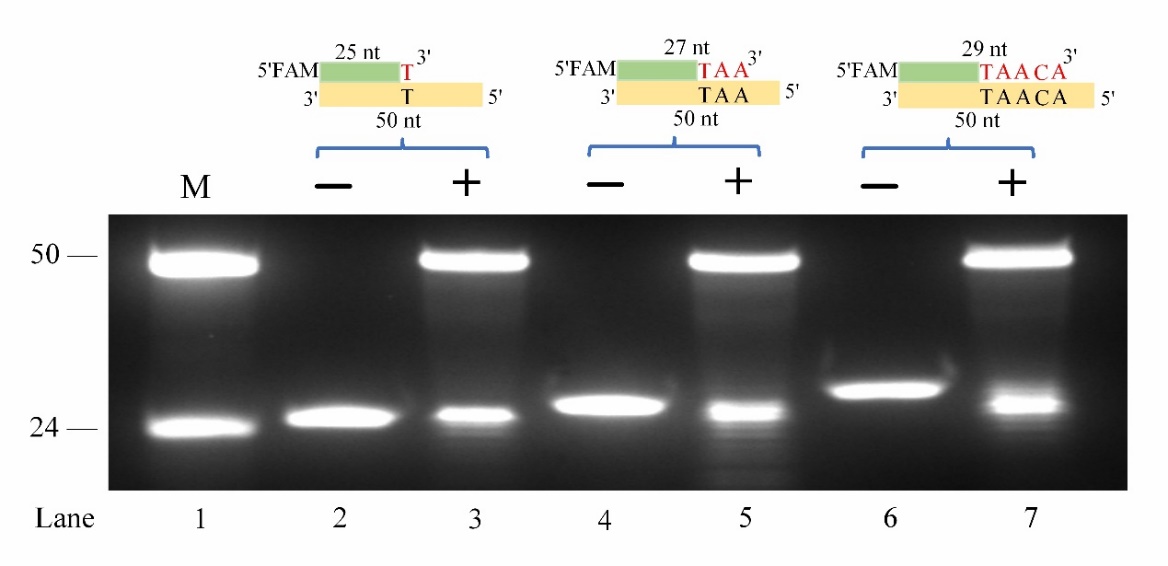


**Additional file 1: Figure S4**. Study on the correction function of IME199 DNAP. IME199 DNAP was incubated with primed-templates mismatched 3′-terminus for 20 min at 30°C and then analyzed on a 20% denaturing PAGE gel. “-” means no IME199 DNAP, and “+” means with IME199 DNAP. The primed-template (25/50 nt) substrate was made by hybridizing two oligonucleotide strands (25 nt oligonucleotide sequence: 5′FAM- TCCTAACGAGATTAGTTTTGCTGTT -3′, 50 nt oligonucleotide sequence 5′- CCCATACAAATAAACCAAAAAACAATACAGCAAAACTAATCTCGTTAGGA -3′). The primed-template (27/50 nt) substrate was made by hybridizing two oligonucleotide strands (27 nt oligonucleotide sequence: 5′FAM- TCCTAACGAGATTAGTTTTGCTGTTAA -3′, 50 nt oligonucleotide sequence 5′- CCCATACAAATAAACCAAAAAACAATACAGCAAAACTAATCTCGTTAGGA -3′). The primed-template (29/50 nt) substrate was made by hybridizing two oligonucleotide strands (29 nt oligonucleotide sequence: 5′FAM- TCCTAACGAGATTAGTTTTGCTGTTAACA -3′, 50 nt oligonucleotide sequence 5′- CCCATACAAATAAACCAAAAAACAATACAGCAAAACTAATCTCGTTAGGA -3′)


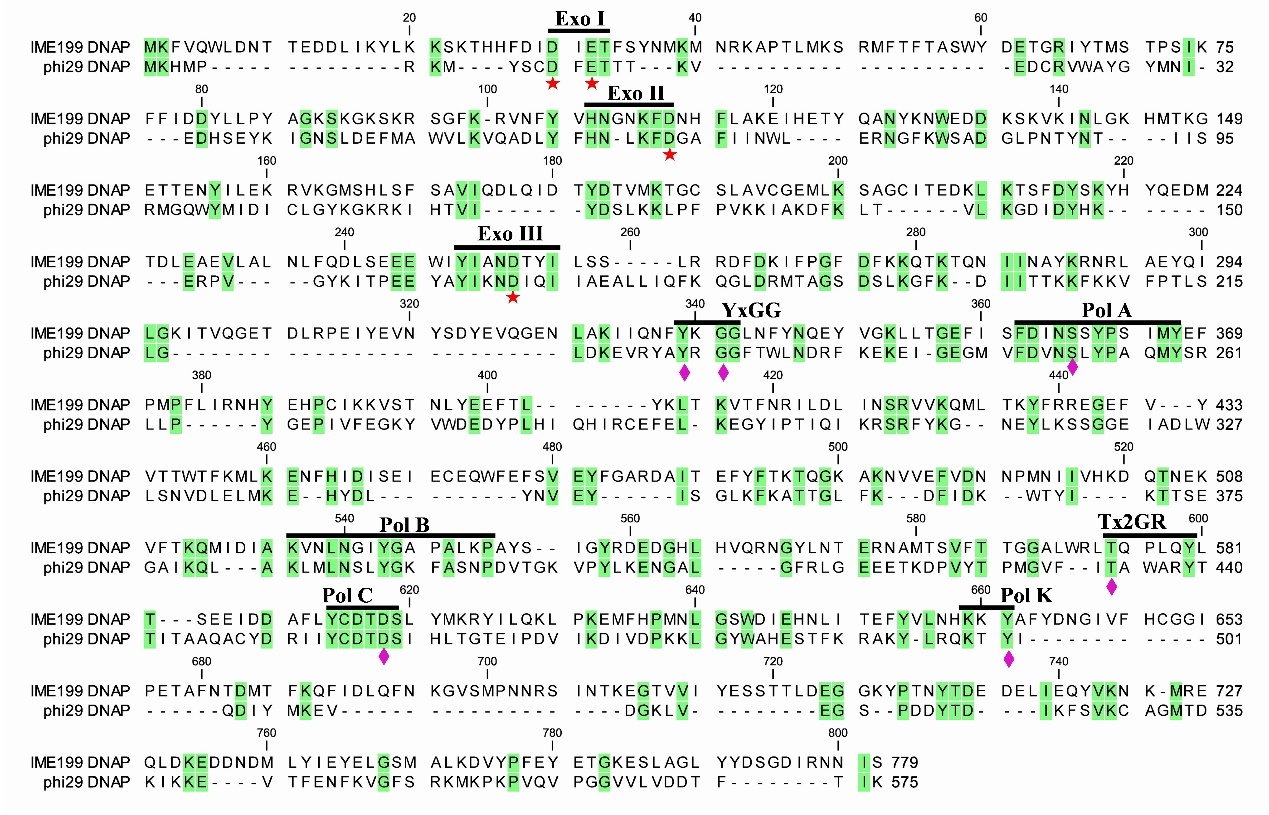


**Additional file 1: Figure S5**. Amino acid sequence analysis between IME199 DNAP and phi29 DNAP. Alignment between IME199 DNAP and phi29 DNAP was made using CLC Sequence Viewer 6, and the regions containing homologous sequences are shown. Numbers indicate amino acid sites. Green color indicates amino acid residues of IME199 DNAP that are similar to those of phi29 DNAP. The conserved motif sequences are underlined in black. Red stars indicate amino acid residues associated with 3'-5' exonuclease activity in phi29 DNAP. Pink diamond indicates amino acid residues associated with polymerase activity in phi29 DNAP

**A**


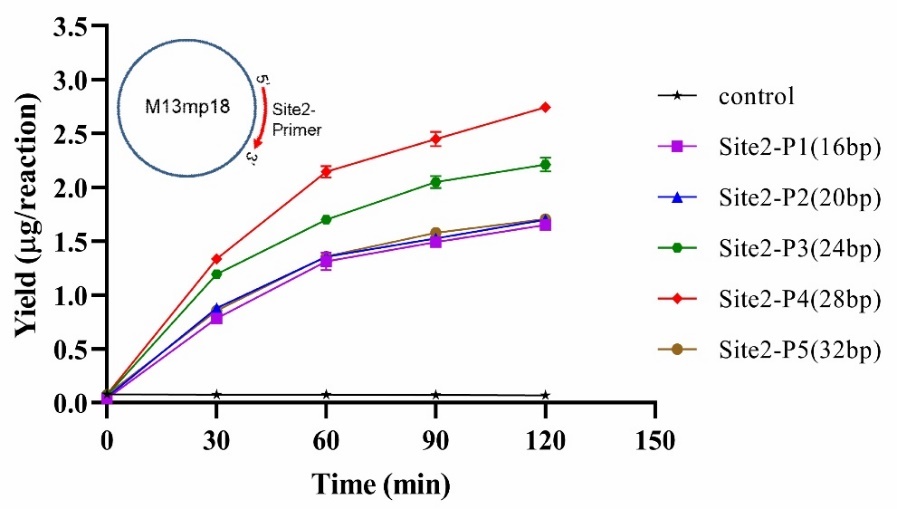


**B**


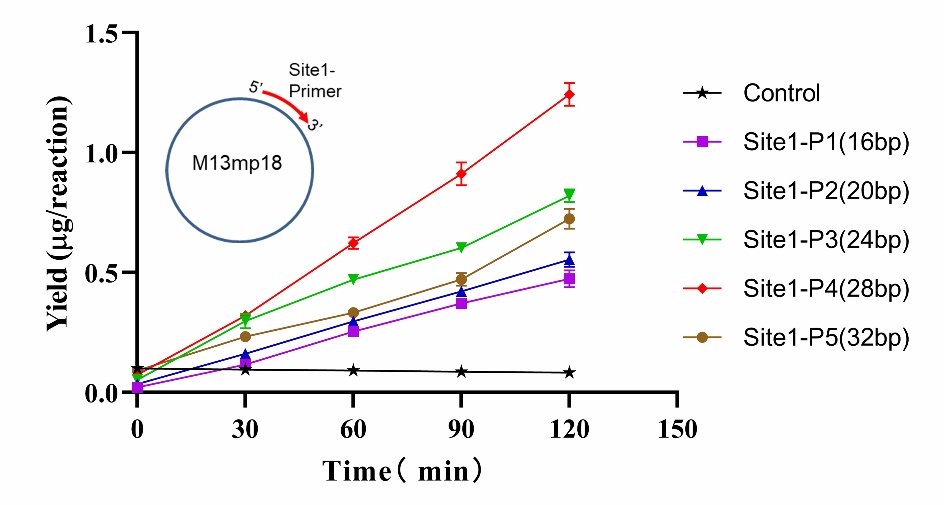


**Additional file 1: Figure S6**. The yield of rolling circle amplification. 25 ng of single-stranded M13mp18 DNA was amplified by 100 nM IME199 DNAP (A) or 10 U phi29 DNAP (B) at 30°C in the presence of primers of different lengths. The yield of DNA was calculated using the equation Yield = v ◊ c, where v is the reaction volume and c is the concentration of detected DNA. Primer sequences are shown in Table S2


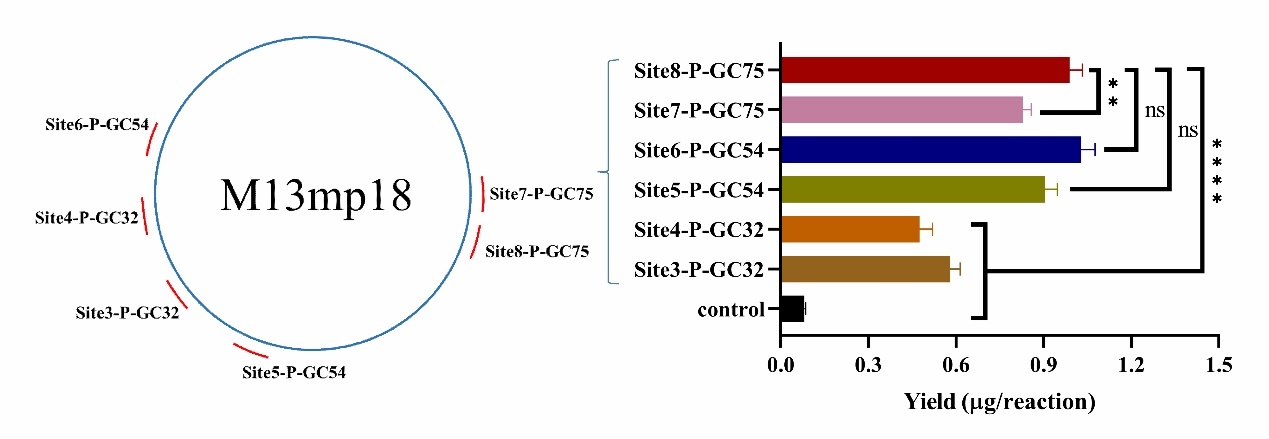


**Additional file 1: Figure S7**. Comparison of phi29 DNAP using 28bp primers with different G+C content for rolling circle amplification. 25 ng of single-stranded M13mp18 DNA was amplified by 10 U phi29 DNAP at 30°C in the presence of primers with different G+C content. The yield of DNA was calculated using the equation Yield = v ◊ c, where v is the reaction volume and c is the concentration of detected DNA. Primer sequences are shown in Supplementary Table S2


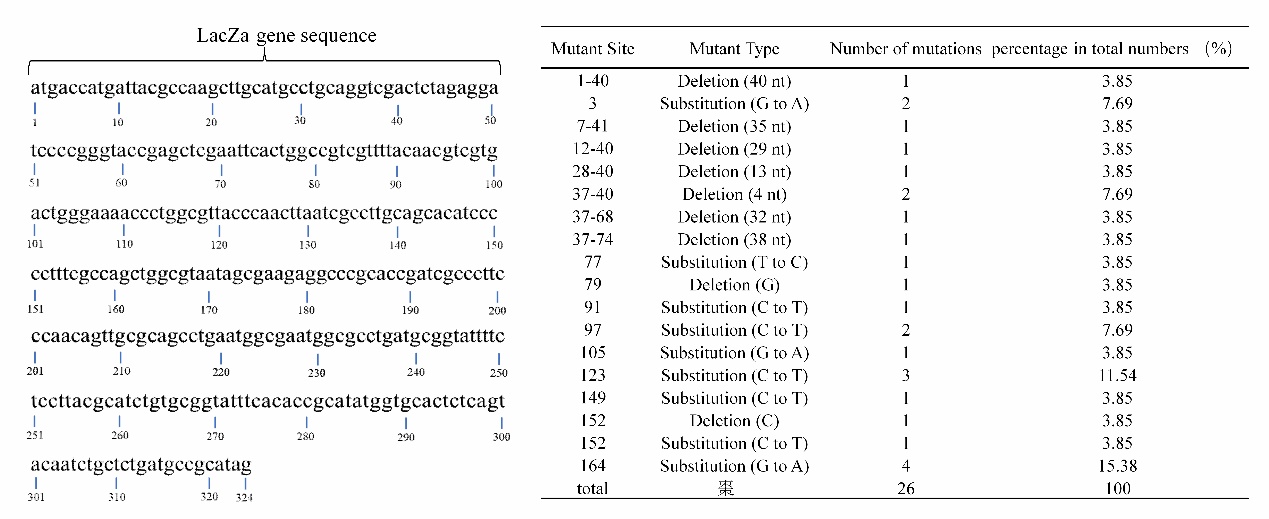


**Additional file 1: Figure S8**. Sequencing results of the lacZα gene on the pUC19 plasmid in the white clones. On the left is the lacZ gene sequence, and the numbers represent the base sites. On the right is a summary of the sequencing results of the pUC19 plasmid in 26 white clones


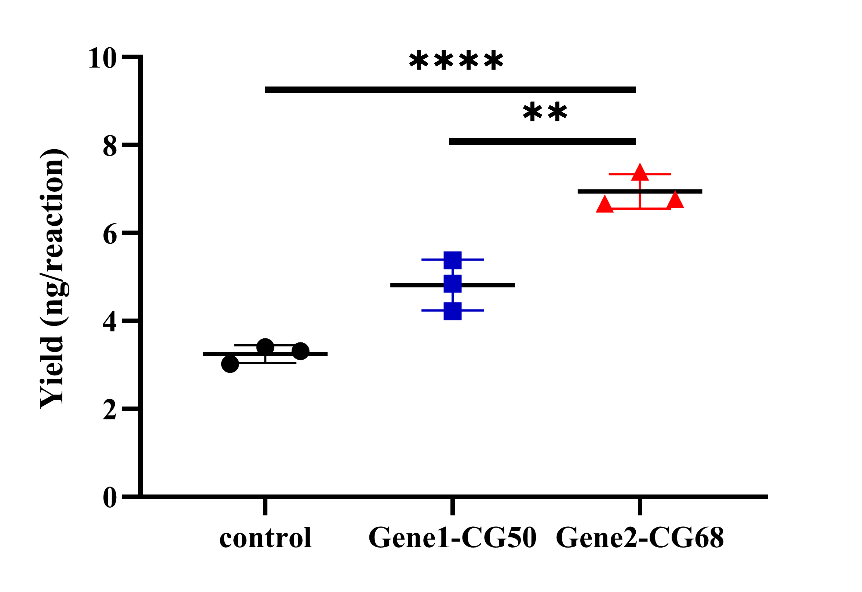


**Additional file 1: Figure S9**. Comparison of IME199 DNAP amplified different G+C sequences. Amplification was performed at 30℃ using random hexamer primers. Gene1 and gene2 are linear double-stranded DNA with a length of about 1600bp

**Additional file 1: Table S1.** Analysis of phage genomes with terminal inverted repeat sequences

| Number | Phage name | Genus | GenBank No. | Genome length（bp） | Reverse repeat  sequence site | Match length（bp） |
| --- | --- | --- | --- | --- | --- | --- |
| 1 | *Enterococcus* phage vB_EfaP_IME199 | *Minhovirus* | KT945995.1 | 18838 | 1-77 vs 18838-18763 | 77 |
| 2 | *Enterococcus* phage vB_EfaP_Zip | *Minhovirus* | MK360025.1 | 18742 | 1-54 vs 18742-18689 | 54 |
| 3 | *Enterococcus* phage vB_EfaP_IME195 | *Copernicusvirus* | KT932700.1 | 18655 | 1-63 vs 18655-18593 | 63 |
| 4 | *Enterococcus* phage Idefix | *Copernicusvirus* | NC_049937.1 | 18168 | 1-64 vs 18168-18105 | 64 |
| 5 | *Enterococcus* phage vB_EfaP_Ef7.2 | *Copernicusvirus* | MK721183.1 | 18737 | 1-52 vs 18719-18667 | 52 |
| 6 | *Enterobacteria* phage PRD1 | *Alphatectivirus* | AY848689.1 | 14927 | 1-110 vs 14927-14818 | 110 |
| 7 | *Enterobacteria* phage PR3 | *Alphatectivirus* | AY848685.1 | 14937 | 1-112 vs 14937-14826 | 112 |
| 8 | *Enterobacteria* phage PR5 | *Alphatectivirus* | AY848687.1 | 14939 | 1-110 vs 14939-14830 | 110 |
| 9 | *Enterobacteria* phage L17 | *Alphatectivirus* | AY848684.1 | 14935 | 1-111 vs 14935-14825 | 111 |
| 10 | *Enterobacteria* phage PR772 | *Alphatectivirus* | AY848688.1 | 14942 | 1-111 vs 14942-14832 | 111 |
| 11 | *Enterobacteria* phage PR4 | *Alphatectivirus* | AY848686.1 | 14954 | 1-111 vs 14954-14844 | 111 |
| 12 | *Staphylococcus* phage 66 | *Rosenblumvirus* | NC_007046.1 | 18199 | 5-220 vs 18197-17983 | 216 |
| 13 | *Staphylococcus* phage vB_SauP_EBHT | *Rosenblumvirus* | MT926124.1 | 17471 | 54-131 vs 17471-17390 | 78 |
| 14 | *Staphylococcus* phage GRCS | *Rosenblumvirus* | KJ210330.1 | 17869 | 1-367 vs 17867-17497 | 367 |
| 15 | *Staphylococcus* phage PSa3 | *Rosenblumvirus* | HF937074.1 | 17602 | 1-237 vs 17601-17375 | 237 |
| 16 | *Staphylococcus* phage S13' | *Rosenblumvirus* | AB626963.1 | 18186 | 1-245 vs 18186-17937 | 245 |
| 17 | *Staphylococcus* virus 44AHJD | *Rosenblumvirus* | AF513032.1 | 16784 | 1-217 vs 16784-16568 | 217 |
| 18 | *Staphylococcus* phage BP39 | *Rosenblumvirus* | KM366100.1 | 17641 | 1-214 vs 17623-17406 | 214 |
| 19 | *Staphylococcus* phage S24-1 | *Rosenblumvirus* | AB626962.1 | 18168 | 1-245 vs 18168-17924 | 245 |
| 20 | *Staphylococcus* phage CSA13 | *Rosenblumvirus* | MH107118.1 | 17034 | 1-234 vs 17034-16801 | 234 |
| 21 | *Staphylococcus* phage phiAGO1.3 | *Rosenblumvirus* | MG766218.1 | 17603 | 1-116 vs 17603-17488 | 116 |
| 22 | *Staphylococcus* phage SLPW | *Rosenblumvirus* | KU992911.1 | 17861 | 1-346 vs 17861-17516 | 346 |
| 23 | *Staphylococcus* phage SAP-2 | *Rosenblumvirus* | EU136189.1 | 17938 | 15-207 vs 17938-17746 | 193 |
| 24 | *Staphylococcus* phage LSA2366 | *Rosenblumvirus* | MW363799.1 | 17056 | 1-177 vs 16977-16805 | 177 |
| 25 | *Staphylococcus* phage SA03-CTH2 | *Rosenblumvirus* | MK936475.1 | 17511 | 44-210 vs 17511-17344 | 167 |
| 26 | *Staphylococcus* phage SA46-CTH2 | *Rosenblumvirus* | MK764384.1 | 17505 | 1-210 vs 17503-17294 | 210 |
| 27 | *Staphylococcus* phage SA1-CTA1 | *Rosenblumvirus* | MK922546.1 | 17527 | 1-211 vs 17513-17303 | 211 |
| 28 | *Streptococcus* phage C1 | *Rosenblumvirus* | NC_004814.1 | 16687 | 1-374 vs 16687-16314 | 374 |
| 29 | *Staphylococcus* phage SA46-CL1 | *Rosenblumvirus* | MK936476.1 | 17508 | 1-211 vs 17503-17293 | 211 |
| 30 | *Staphylococcus* phage St 134 | *Andhravirus* | KY471386.1 | 18275 | 24-146 vs 18274-18150 | 123 |
| 31 | *Staphylococcus* phage Pike | *Andhravirus* | MH972261.1 | 18376 | 1-149 vs 18376-18228 | 149 |
| 32 | *Staphylococcus* phage Andhra | *Andhravirus* | KY442063.1 | 18546 | 8-198 vs 18540-18350 | 191 |
| 33 | *Streptococcus* phage CP-7 | *Cepunavirus* | LK392619.1 | 19741 | 1-378 vs 19741-19364 | 378 |
| 34 | *Streptococcus* phage Cp1 | *Cepunavirus* | NC_001825.1 | 19343 | 1-352 vs 19343-18992 | 352 |
| 35 | *Bacillus* phage phi29 | *Salasvirus* | NC_011048 | 19282 | 1-6 vs 19282-19277 | 6 |
| 36 | *Bacillus* phage Whiting18 | *Salasvirus* | MW477480.1 | 19548 | 1-34 vs 19430-19397 | 34 |
| 37 | *Bacillus* phage vB_BveP-Goe6 | *Salasvirus* | MF407276.1 | 19105 | 1-6 vs 19105-19100 | 6 |
| 38 | *Bacillus* phage Gxv1 | *Salasvirus* | MT459794.1 | 21781 | 1-6 vs 21781-21776 | 6 |
| 39 | *Bacillus* phage Nf | *Beecentumtrevirus* | EU622808.1 | 18753 | 1-8 vs 18753-18746 | 8 |
| 40 | *Bacillus* phage B103 | *Beecentumtrevirus* | NC_004165.1 | 18630 | 1-6 vs 18630-18625 | 6 |
| 41 | *Bacillus* phage vB_BsuP-Goe1 | *Beecentumtrevirus* | NC_049975.1 | 18379 | 1-6 vs 18379-18374 | 6 |
| 42 | *Bacillus* phage GA-1 | *Gaunavirus* | NC_002649.1 | 21129 | 1-7 vs 21129-21123 | 7 |
| 43 | *Bacillus* phage SRT01hs | *Gaunavirus* | MN857617.1 | 20784 | 1-6 vs 20784-20779 | 6 |
| 44 | *Bacillus* phage AP50 | *Betatectivirus* | EU408779.1 | 14398 | 1-27 vs 14398-14372 | 27 |
| 45 | *Bacillus* phage pGIL02 | *Betatectivirus* | CP013282.1 | 14961 | 2-75 vs 14934-14862 | 74 |
| 46 | *Bacillus* phage Bam35c | *Betatectivirus* | NC_005258.1 | 14935 | 1-74 vs 14935-14862 | 74 |
| 47 | *Bacillus* phage BeachBum | *Harambevirus* | KY921761.1 | 21054 | 1-52 vs 21054-21003 | 52 |
| 48 | *Bacillus* phage Harambe | *Harambevirus* | KY821088.1 | 21684 | 1-52 vs 21684-21634 | 52 |
| 49 | *Bacillus* phage vB_Bpu_PumA1 | *Bundooravirus* | MN524844.1 | 18466 | 1-11 vs 18466-18456 | 11 |
| 50 | *Bacillus* phage vB_Bpu_PumA2 | *Bundooravirus* | MN524845.1 | 18932 | 1-11 vs 18932-18922 | 11 |
| 51 | *Bacillus* phage WhyPhy | *Bundooravirus* | MW419775.1 | 18642 | 1-12 vs 18642-18631 | 12 |
| 52 | *Bacillus* phage QCM11 | *Claudivirus* | KX961631.1 | 26054 | 2-8 vs 26054-26048 | 7 |
| 53 | *Bacillus* phage vB_BthP-Goe4 | *Claudivirus* | MH817022.1 | 25722 | 1-8 vs 25722-25715 | 8 |
| 54 | *Bacillus* phage SerPounce | *Claudivirus* | KY947509.1 | 27206 | 1-8 vs 27206-27199 | 8 |
| 55 | *Bacillus* phage Stitch | *Claudivirus* | KX349901.1 | 24320 | 2-7 vs 24320-24315 | 6 |
| 56 | *Bacillus* phage Thornton | *Claudivirus* | MW348917.1 | 26319 | 1-8 vs 26319-26312 | 8 |
| 57 | *Bacillus* phage DLc1 | *Huangshavirus* | MW012634.1 | 28950 | 1-5 vs 28950-28946 | 5 |
| 58 | *Bacillus* phage DK1 | *Hemphillvirus* | MK284526.1 | 27180 | 1-9 vs 27180-27172 | 9 |
| 59 | *Bacillus* phage DK2 | *Hemphillvirus* | MK284527.1 | 26357 | 1-7 vs 26357-26351 | 7 |
| 60 | *Bacillus* phage DK3 | *Hemphillvirus* | MK284528.1 | 26865 | 1-10 vs 26865-26856 | 10 |
| 61 | *Bacillus* phage MG-B1 | *Klosterneuburgvirus* | KC685370.1 | 27190 | 1-22 vs 27190-27169 | 22 |
| 62 | *Rhizobium* phage RHph_N3_8 | *Caudoviricetes* | MN988551.1 | 18277 | 4-49 vs 18277-18232 | 46 |
| 63 | *Clostridium* phage susfortuna | *Susfortunavirus* | MH393889.1 | 19046 | 1-76 vs 19046-18971 | 76 |
| 64 | *Clostridium* phage CPD7 | *Susfortunavirus* | MK017820.1 | 18958 | 51-108 vs 18941-18884 | 58 |
| 65 | *Clostridium* phage CPS2 | *Brucesealvirus* | MH248069.1 | 17961 | 1-366 vs 17961-17596 | 366 |
| 66 | *Clostridium* phage phiZP2 | *Brucesealvirus* | JQ729992.1 | 18078 | 1-79 vs 18078-17988 | 79 |
| 67 | *Gluconobacter* phage GC1 | *Gammatectivirus* | MG159787.1 | 16532 | 1-326 vs 16532-16198 | 326 |
| 68 | *Lactococcus* phage WP-2 | *Negarvirus* | KJ528544.1 | 18899 | 3-166 vs 18897-18734 | 134 |
| 69 | *Actinomyces* phage Av-1 | *Dybvigvirus* | DQ123818.2 | 17171 | 1-210 vs 17171-16962 | 210 |
| 70 | *Streptococcus* phage SOCP | *Cepunavirus* | KJ617393.1 | 19347 | 1-352 vs 19347-18996 | 352 |
| 71 | *Mycoplasma* virus P1 | *Delislevirus* | NC_002515.1 | 11660 | 1-350 vs 11660-11311 | 350 |

**Additional file 1: Table S2:** Sequences of the oligonucleotides that have been used in this study.

| Name | Sequence (5’→3’) | Length (nt) |
| --- | --- | --- |
| IME199 DNAP-F | cgggatccATGAAATTCGTACAATGGTTAGACAAT | 35 |
| IME199 DNAP-R | ccgctcgagTCAAGAAATATTATTTCTAATATCTCCTGAA | 40 |
| ENC-1 | (FAM)TCCTAACGAGATTAGTTTTGCTGT | 24 |
| ENC-2 | TCCTAACGAGATTAGTTTTGCTGT(FAM) | 24 |
| PLM-F1 | (FAM)TCCTAACGAGATTAGTTTTGCTGTATTGTTTTTT  GGTTTATTTGTATGGG | 50 |
| PLM-R | CCCATACAAATAAACCAAAAAACAATACAGCAAAA  CTAATCTCGTTAGGA | 50 |
| PLM-F2 | (FAM)TCCTAACGAGATTAGTTTTGCTGTT | 25 |
| PLM-F3 | (FAM)TCCTAACGAGATTAGTTTTGCTGTTAA | 27 |
| PLM-F4 | (FAM)TCCTAACGAGATTAGTTTTGCTGTTAACA | 29 |
| D30A-F | GCACCATTTTGATATAGCTATTGAAACT | 28 |
| D30A-R | GCTATATCAAAATGGTGCGTTTTTGA | 26 |
| E32A-F | TGATATAGATATTGCAACTTTTTC | 24 |
| E32A-R | GCAATATCTATATCAAAATGGTGC | 24 |
| D112A-F | GGAAATAAGTTTGCCAATCACTTTC | 25 |
| D112A-R | GCAAACTTATTTCCATTATGAACG | 24 |
| D251A-F | GGATTTACATTGCTAATGCCACATATATTC | 30 |
| D251A-R | GCATTAGCAATGTAAATCCACTCTTCTTC | 29 |
| Y333A-F | ATCATTCAGAACTTTGCCAAAGGTGGAC | 28 |
| Y333A-R | GCAAAGTTCTGAATGATTTTAGCTAGA | 27 |
| G335A-F | CAGAACTTTTACAAAGCTGGACTTAAT | 27 |
| G335A-R | GCTTTGTAAAAGTTCTGAATGATTTTAGC | 29 |
| S360A-F | CAGTTTTGATATTAACGCTTCTTATCCCTC | 30 |
| S360A-R | GCGTTAATATCAAAACTGATAAACTCTCCGG | 31 |
| T575A-F | GGTGCTTTATGGAGGTTAGCTCAACCATT | 29 |
| T575A-R | CTAACCTCCATAAAGCACCTCCTGTTGTA | 29 |
| D596A-F | TATATTGTGATACAGCTAGTCTTTAT | 26 |
| D596A-R | GCTGTATCACAATATAAAAAAGCGTC | 26 |
| Site3-P-GC32 | AAATCGGTTGTACCAAAAACATTATGAC | 28 |
| Site4-P-GC32 | TTAGAGAGTACCTTTAATTGCTCCTTTT | 28 |
| Site5-P-GC54 | GAAACCAGGCAAAGCGCCATTCGCCATT | 28 |
| Site6-P-GC54 | GATGAACGGTGTACAGACCAGGCGCATA | 28 |
| Site7-P-GC75 | CTGCGCGTAACCACCACACCCGCCGCGC | 28 |
| Site8-P-GC75 | CGAAAGGAGCGGGCGCTAGGGCGCTGGC | 28 |
| Site1-P1 | TCATAGCTGTTTCCTG | 16 |
| Site1-P2 | ATGGTCATAGCTGTTTCCTG | 20 |
| Site1-P3 | AATCATGGTCATAGCTGTTTCCTG | 24 |
| Site1-P4 | TCGTAATCATGGTCATAGCTGTTTCCTG | 28 |
| Site1-P5 | TCGTAATCATGGTCATAGCTGTTTCCTGTGTG | 30 |
| Site2-P1 | ACCACCACACCCGCCG | 16 |
| Site2-P2 | AACCACCACACCCGCCGCGC | 20 |
| Site2-P3 | GCGCGTAACCACCACACCCGCCGC | 24 |
| Site2-P4 | CTGCGCGTAACCACCACACCCGCCGCGC | 28 |
| Site2-P5 | CACGCTGCGCGTAACCACCACACCCGCCGCGC | 30 |

Lowercase bases represent restriction sites.

The substitution bases are underlined.

**References**

[1] Katoh K, Rozewicki J, Yamada KD. MAFFT online service: multiple sequence alignment, interactive sequence choice and visualization. Brief Bioinform. 2019. 20(4): 1160-1166.

[2] Gouet P, Robert X, Courcelle E. ESPript/ENDscript: Extracting and rendering sequence and 3D information from atomic structures of proteins. Nucleic Acids Res. 2003. 31(13): 3320-3.
